# Correlation between the genomic *o454-nlpD* region polymorphisms, virulence gene equipment and phylogenetic group of extraintestinal *Escherichia coli* (ExPEC) enables pathotyping irrespective of host, disease and source of isolation

**DOI:** 10.1186/s13099-014-0037-x

**Published:** 2014-09-16

**Authors:** Christa Ewers, Flavia Dematheis, Haritha Devi Singamaneni, Nishant Nandanwar, Angelika Fruth, Ines Diehl, Torsten Semmler, Lothar H Wieler

**Affiliations:** 1 Institute for Hygiene and Infectious Diseases of Animals, Justus-Liebig-Universität Giessen, Frankfurter Str. 85-89, Giessen, 35392, Germany; 2 Centre for Infection Medicine, Institute of Microbiology and Epizootics, Freie Universität Berlin, Robert-von-Ostertag-Str. 7-13, Berlin, 14163, Germany; 3 Pathogen Biology Laboratory, Department of Biotechnology and Bioinformatics, University of Hyderabad, Hyderabad 500046, Gachibowli, India; 4 National Reference Centre for Salmonella and Other Enteric Pathogens, Robert Koch Institute, Burgstr. 37, Wernigerode, 38855, Germany

**Keywords:** Escherichia coli, MLST, Ecor, mutS-rpoS and o454-nlpD genomic regions

## Abstract

**Background:**

The *mutS*-*rpoS* intergenic region in *E. coli* displays a mosaic structure which revealed pathotype specific patterns. To assess the importance of this region as a surrogate marker for the identification of highly virulent extraintestinal pathogenic *E. coli* (ExPEC) strains we aimed to: (i) characterize the genetic diversity of the *mutS* gene and the *o454-nlpD* genomic region among 510 *E. coli* strains from animals and humans; (ii) delineate associations between the polymorphism of this region and features such as phylogenetic background of *E. coli*, pathotype, host species, clinical condition, serogroup and virulence associated genes (VAG)s; and (iii) identify the most important VAGs for classification of the *o454-nlpD* region.

**Methods:**

Size variation in the *o454-nlpD* region was investigated by PCR amplification and sequencing. Phylogenetic relationships were assessed by Ecor- and Multilocus sequence- typing (MLST), and a comparative analysis between *mutS* gene phylogenetic tree obtained with RAxML and the MLST grouping method was performed. Correlation between *o454-nlpD* patterns and the features described above were analysed. In addition, the importance of 47 PCR-amplified ExPEC-related VAGs for classification of *o454-nlpD* patterns was investigated by means of Random Forest algorithm.

**Results:**

Four main structures (patterns I-IV) of the *o454-nlpD* region among ExPEC and commensal *E. coli* strains were identified. Statistical analysis showed a positive and exclusive association between pattern III and the ExPEC strains. A strong association between pattern III and either the Ecor group B2 or the sequence type complexes known to represent the phylogenetic background of highly virulent ExPEC strains (such as STC95, STC73 and STC131) was found as well. RF analyses determined five genes (*csgA*, *malX*, *chuA*, *sit*, and *vat*) to be suitable to predict pattern III strains.

**Conclusion:**

The significant association between pattern III and group B2 strains suggested the *o454-nlpD* region to be of great value in identifying highly virulent strains among the mixed population of *E. coli* promising to be the basis of a future typing tool for ExPEC and their gut reservoir. Furthermore, top-ranked VAGs for classification and prediction of pattern III were identified. These data are most valuable for defining ExPEC pathotype in future *in vivo* assays.

## 1 Background

*Escherichia coli* is a normal inhabitant of the gastrointestinal microbiota of mammalians and birds, but at the same time it can cause a variety of diseases relevant for public and animal health such as diarrhoea, bacteraemia, septicaemia, urinary tract infections [1]. From a clinical perspective, *E. coli* is broadly classified into commensals, intestinal pathogenic *E. coli* (InPEC) and extraintestinal pathogenic *E. coli* (ExPEC), the latter group being further divided into uropathogenic *E. coli* (UPEC), septicaemia-associated *E. coli* (SEPEC), neonatal meningitis *E. coli* (NMEC), and avian pathogenic *E. coli* (APEC). ExPEC strains are normal colonizers of the gut of men and animals, but in contrast to intestinal pathogenic variants, they can cause infections to the urinary tract or the blood stream [2] once they reach the corresponding body site. Although collectively termed ExPEC, simply to reflect their shared ability to express functionally similar virulence factors and to denote considerable overlaps concerning serotypes and phylogenetic background [3],[4], this group of strains exhibits large genome diversity. This has been mainly attributed to the frequent location of virulence associated genes (VAGs) on plasmids, pathogenicity islands, or phages, allowing the VAGs to be highly interchangeable among strains through horizontal gene transfer (HGT) [5],[6]. The population structure of *E. coli* is characterised by the presence of distinct phylogenetic groups as observed by phylogenetic reconstruction [7],[8] or by the use of specific markers [9]. Based on these approaches, four (A, B1, B2 and D) major phylogroups have been described while according to the method, two minor (E and F) or two hybrid (AxB1 and ABD) phylogroups have been defined in addition, which are not necessarily equivalent [8]–[10]. The distribution (presence/absence) of virulence factors thought to be involved in the ability of a strain to cause diverse diseases also varies among strains of these phylogenetic groups, indicating a role of the genetic background in the expression of virulence [11]–[13].

The high diversity of ExPEC and the difficulty in a clear demarcation of these facultative pathogenic strains from their commensal counterpart poses a huge challenge to infectious medicine in terms of diagnostic and risk assessment. As recently shown, the genetic variability of the *mutS*-*rpoS* chromosomal region may serve as indicator and thus as a chromosomal marker for the different virulence potential of *E. coli* strains [2],[14]–[16]. The crucial genes are *mutS*, which encodes one of the four proteins required for methyl-directed mismatch repair (MMR) of DNA, and *rpoS*, which encodes a sigma factor (sigma 38) that regulates many stationary-phase and environmental stress response genes [17]. Although *mutS* and *rpoS* are generally conserved in Enterobacteriaceae, the *mutS-rpoS* intergenic and its adjacent region revealed extensive genetic variability that was subjected to genetic exchange during the evolution of pathogenic lineages. Several studies revealed a pathotype-associated polymorphism in this genetic region [15],[18],[19] suggesting it to be the region owing to HGT and evolutionary processes. In comparison to *E. coli* K-12, previous studies revealed that enteropathogenic *E. coli* (EPEC), enterohaemorrhagic *E. coli* (EHEC) and *E. coli* group B2 strains harbour specific DNA insertions within the *mutS-rpoS* intergenic region [15],[16],[19]. An insertion of 2.1 kb, in place of the initially identified 2.9 kb insert at the proximity of *E. coli* O157:H7 [19] has been found in strains of uropathogenic *E. coli*[15] and larger intergenic regions exist in strains of EPEC and EHEC [16]. Moreover, phylogenetic analysis of EHEC and EPEC strains, as well as strains of the Ecor collection, revealed that the *mutS* gene itself may be frequently subject to horizontal transfer and recombination during the evolution of these strains which is consistent with mechanism for stabilizing adaptive changes promoted by *mutS* mutators with relaxed recombination barriers [14],[20].

In the present study we attempted to assess the importance of the *mutS-rpoS* intergenic region as a surrogate marker for the rapid identification of highly virulent ExPEC strains and to delineate biological meaningful subgroups among this highly diverse group of strains. In particular, we aimed: (i) to characterize the genetic diversity of the *mutS* gene and of the *o454-nlpD* genomic region among 510 *E. coli* strains obtained from animal and human sources; (ii) to delineate associations between the polymorphism of this region and features such as phylogenetic background of *E. coli*, bacterial class or pathotype, host species, clinical condition, serogroup and virulence associated genes (VAG)s; and (iii) to identify the most important ExPEC-related VAGs for classification of the *o454-nlpD* genomic region by using the random forest (RF) algorithm [21]. The results could be a valuable contribution to ongoing analyses on pathoadaptive alterations in ExPEC strains that affect disease severity and may have consequences for diagnostics of *E. coli* infections (15).

Here we provide sound evidence that this polymorphic region indeed is correlated with virulence, and with the high number of newly generated whole genome data of *E. coli*, researchers working with this versatile bacterial pathogen can further proof validity of our data in a larger context.

## 2 Results

### 2.1 Size variation and DNA polymorphism in the fhlA-nlpD region with respect to the phylogenetic background of E. coli strains

PCR amplification of the *o454-nlpD* region among 510 *E. coli* strains revealed a mosaic structure of the *o454-nlpD* genomic region. Four patterns characterized by a differentiating length and genomic structure, were identified: pattern I was defined by the absence of a PCR product due to a lack of *o454* gene (synonyms *b2740* and *ygbN*), which codes for a putative Zn^++^-dependent hydrolase and a permease; pattern II showed an amplicon of 1.319 bp in length consisting of the full sequence of the RNA-polymerase gene *rpoS*; pattern III consisted of a DNA fragment of 3.685 bp in length carrying the full sequences of hypothetical protein genes *c3302*, *c3303* and *c3304* genes; pattern IV comprised a DNA fragment of 4.546 bp in length carrying the full gene sequences of hydroxybenzoate decarboxylase subunit genes *kpdD*, *kpdC*, *kpdB* and of Salmonella lysis gene *slyA* (transcriptional regulator gene, required for survival within macrophages). Only exceptionally, other amplicon sizes were observed. One human commensal strain (IMT13844; ST405) showed an *o454-nlpD* pattern of ca. 2000 bp length. Sequencing of this DNA region revealed the insertion of a mobile element between *o454* and *rpoS* sharing 100% identity with a transposase of the IS*200* family [22]. Strain Ecor6 principally showed MG1655-specific pattern I, but with a 354 bp-deletion in the *rpoS* gene (position 298 to 651 bp). Strain Ecor32 revealed pattern III with a ca. 300 bp deletion and Ecor36 showed a pattern IV structure with a ca. 250 bp insertion of unknown sequence.

**Figure 1:**
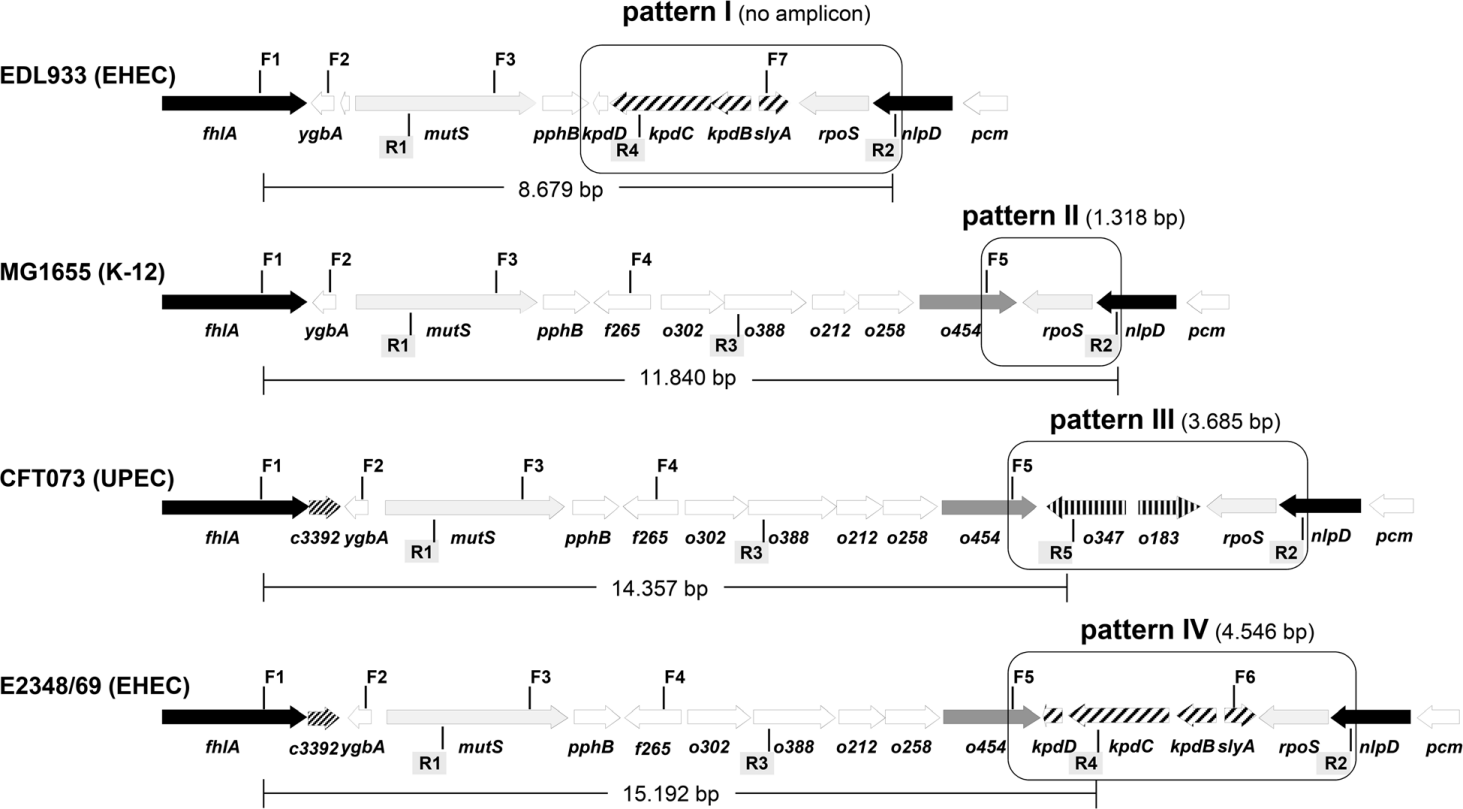
**Physical maps of the** ***fhlA-nlpD*** **intergenic region of** ***E. coli*** **strains as determined by publicly available sequences (EDL933, GenBank NC_002655; MG1655, U00096; CFT073, AE014075; E2348/69, FM180568), showing positions of ORFs and location of** ***o454-nlpD*** **pattern sequences and sizes amplified by PCR; the position of oligonucleotide primers (F = forward; R = reverse) are indicated and the length of fragments amplified from different strains and primer combinations are provided in Table**1**.**

The results of pattern distribution were in agreement with the presence/absence and sizes of other amplicons along the *fhlA*-*nlpD* genetic region (Table 1 and Figure 1). For example, *fhlA*-*mutS* amplicons (primers F1/R1; Table 1) of 1.673 bp and *o454*-*o347* amplicons (primers F5/R5) of 816 bp in size were exclusively determined in pattern III strains, such as UPEC strain CFT073. A 716-bp *o454*-*yclC* (*kpdC*) amplicon (primers F5/R4) was only detected in pattern IV strains, and a 1318-bp *mutS*-*yclC*/*kpdC* amplicon was only present in pattern I strains.

**Table 1:** **Oligonucleotide primers used for the amplification of the intergenic and adjacent** ***o454-nlpD*** **genomic region of** ***E. coli***

**Primer name**		**Genetic region**^**a**^	**Predicted amplicon size (bp) in reference strains**^**b**^				**Forward/reverse primer sequences (5′-3′)**
**For**	**Rev**		**MG1655**	**CFT073**	**E2348/69**	**EDL933**	
F1	R1	*fhlA-mutS*	1.208	1.673	1.208	1.208	AGAGTTCCGTAGCGATCTC/CTGCTGCATCATGGGCGTAT
F1	R2	*fhlA-nlpD*	11.840	14.357	15.192	8.679	AGAGTTCCGTAGCGATCTC/ CATAACGACACAATGCTGGTCC
F2	R1	*ygbA-mutS*	361	360	359	361	TTCACGAGAGATACGCTTGC/CTGCTGCATCATGGGCGTAT
F3	R3	*mutS-o388*	2.881	2.881	2.881	-	AAAGCATTTCGCCGAACGCCGC/CATCGGCGATAACGCCAAT
F3	R4	*mutS-yclC*	-	-	7.365	1.318	AAAGCATTTCGCCGAACGCCGC/GACAACCGTGGTCACTACA
F4	R3	*f265-o388*	1.146	1.146	1.146	-	ACGCTCTACGGGTATCAACT/CATCGGCGATAACGCCAAT
F5	R2	*o454-nlpD*	1.318	3.685	4.546	-	AAGCCCTTGCCAACATGCTAC/CATAACGACACAATGCTGGTCC
F5	R5	*o454-o347*	-	816	-	-	AAGCCCTTGCCAACATGCTAC/CGTCAGGTTGAAATGCTTGACT
F5	R4	*o454-yclC*	-	-	716	-	AAGCCCTTGCCAACATGCTAC/GACAACCGTGGTCACTACA
F6	R2	*slyA-nlpD*	-	-	1.394	1.393	TTGAGTGCAGAAGAGCAGG/CATAACGACACAATGCTGGTCC
F2	R1	*c3292-mutS*	-	1.120	1.119	-	TAACCGGAACAGTTAGCGC/CTGCTGCATCATGGGCGTAT

^a^Biological functions of amplified genes: *fhlA*, formate hydgrogen lyase; *mutS*, mismatch repair protein; *nlpD*, outer membrane lipoprotein; *ygbA*, hypothetical protein; *o388* (syn. *ygbK*), hypothetical protein; *yclC* (syn. *kpdC*), 4-hydroxybenzoate decarboxylase determinant; *f265* (syn. *ygbL*), hypothetical transcriptional regulator; *o454* (syn. *b2740* and *ygbN*), putative zinc-dependent hydrolase; *o347* (syn. *c3302*), limited level of similarity to enzymes implicated in antibiotic hydrolysis and synthesis; *slyA* (syn. *kpdR*), 4-hydroxybenzoate decarboxylase determinant, in *Salmonella* transcriptional regulator, required for survival within macrophages the induction of hemolytic and pore-forming proteins; *c3292*, putative molybdenum-pterin-binding protein. ^b^Based on whole genome sequences deposited to GenBank under accession numbers U00096 (MG1655), NC_004431 (CFT073), FM180568 (E2348/69), and NC_0022655 (EDL933).

**Figure 2:**
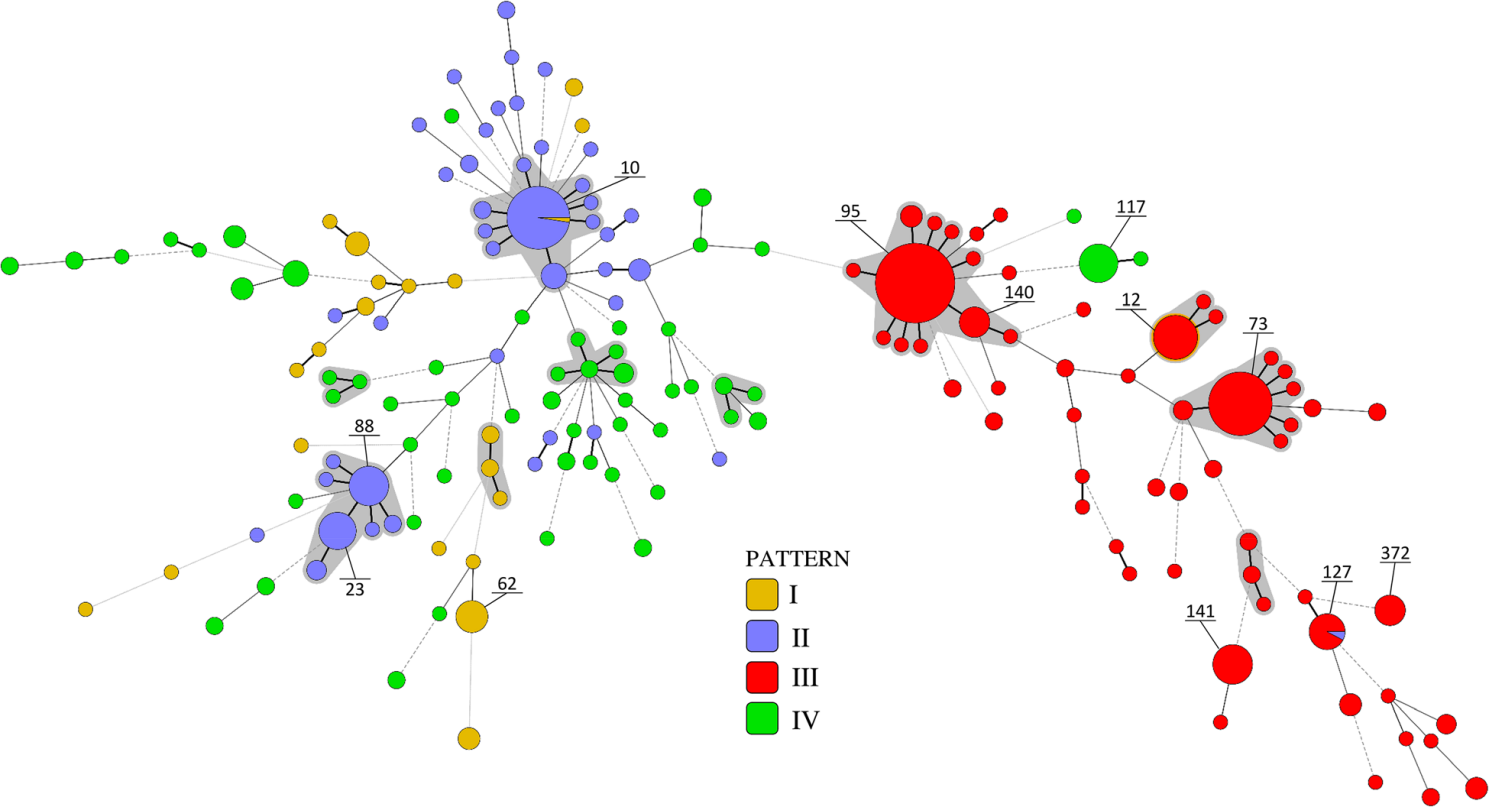
**Minimum spanning tree depicting the distribution of*o454-nlpD*patterns I to IV of extraintestinal (n = 367) and commensal (n = 143)*E. coli*strains in their phylogenetic background, as determined by MLST.** Sequence types (STs) are indicated by numbers.

To link the different *o454-nlpD* regions with the phylogenetic background of the strains, multilocus sequence- typing (MLST) and phylogenetic grouping by structure analysis was performed. MLST revealed that the *E. coli* strains under study were dispersed among 173 STs, and 54 of them were allocated to the following ST complexes: STC73, STC14, STC12, STC95, STC101, SC155, STC10, STC350, STC86 and STC23. The most prominent STCs, harbouring half of the strains investigated, were STC95, STC10, STC73, STC23, and STC12. *E. coli* strains carrying the *o454-nlpD* fragment of 3.656 bp (pattern III) were nearly exclusively allocated to Ecor group B2-ST complexes such as STC95, STC73 and STC12 as shown in Figure 2. Generally, the strains were classified into six phylogenetic groups including hybrid groups ABD and AxB1 as follows: A (n = 71), ABD (n = 53), AxB1 (n = 39), B1 (n = 60), B2 (n = 250) and D (n = 37). *E. coli* strains characterized by pattern III were found exclusively in B2 group with only two exceptions (Table 2).

**Table 2:** **Distribution of** ***o454-nlpD*** **patterns with respect to** ***E. coli*** phylogenetic groups*

	**Pattern I**	**Pattern II**	**Pattern III**	**Pattern IV**
**ExPEC (n = 367)**				
A	1	35	0	1
ABD	18	2	2	21
AxB1	1	6	0	13
B1	0	37	0	15
B2	0	1	196	0
D	7	0	0	11
**Fecal (n = 143)**				
A	0	34	0	0
ABD	6	1	0	3
AxB1	1	4	0	14
B1	0	2	0	0
B2	0	0	53	0
D	7	2	0	10
**Total No. of strains (n = 510)**				
A	1	69	0	1
ABD	24	3	2	24
AxB1	2	10	0	27
B1	0	39	0	21
B2	0	1	249	0
D	14	2	0	21

*o454-nlpD* patterns: I = *o454*-negative, II = 1.319 bp, III = 3.685 bp, IV = 4.546 bp.

*One *E. coli* strain which carries the *IS*2000 in the *o454-nlpD* region is not included here.

**Figure 3:**
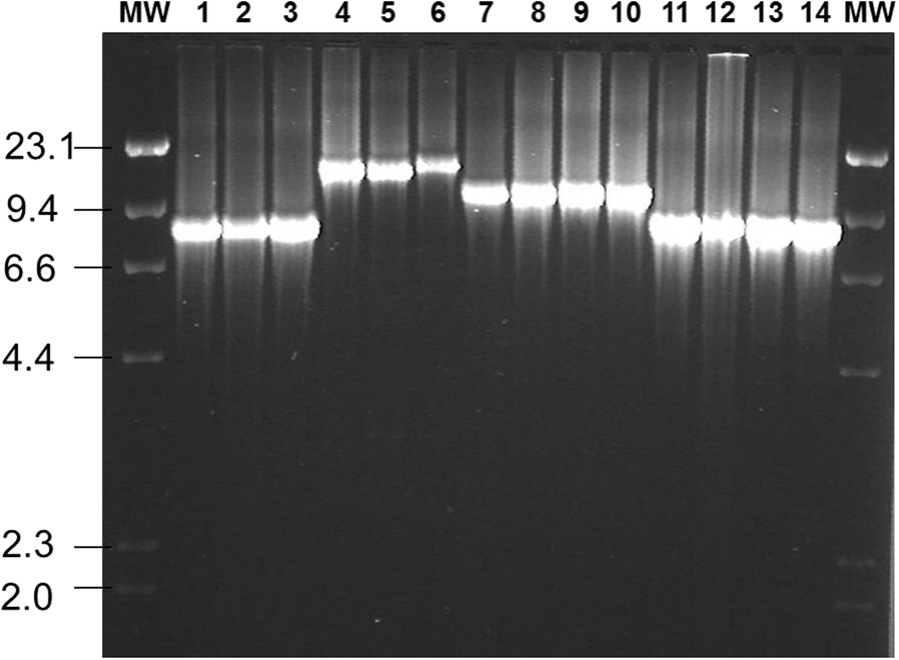
**Resolution of long range PCR amplicons corresponding to the genomic region extending from*fhlA*to*nlpD*.** MW = molecular weight marker (Lambda DNA-Hind III ladder), 1. DEC 4a (EHEC), 2. DEC 3c (EHEC), 3. EDL933 (EHEC), 4. IMT9582 (APEC), 5. IMT7847 (UPEC), 6. V-2 (APEC), 7. IMT10718 (Avian commensal), 8. IMT9238 (APEC), 9. E291 (Human commensal), 10. E291 (UPEC), 11. EC10 (NMEC); 12. F645 (SEPEC), 13. IMT11085 (NMEC), 14. VE239/94 (NMEC).

The genomic region surrounding the *mutS*-*rpoS* genes was further amplified from *E. coli* strains EDL933, MG1655, CFT073, and E2348/69 as a single ~ 8.6 kb, 11.8 kb, 14.3 kb, and 15.2 kb fragment, respectively, as predicted from the genomic sequence (Figure 1). Application of the long range PCR to a subset of 225 strains of *E. coli* representing different hosts, clinical origins and phylogenetic groups also ranged in size between ~ 8 kb and 15 kb (Figure 3). Digestion of the long PCR amplicons with the four-base cutting enzyme AluI resolves the 225 strains into 19 different RFLP types (types A to S) with an RFLP type defined as revealing a unique restriction profile and containing at least 2 strains. In addition, eight singletons have been determined and RFLP types are exemplarily shown in an additional File (see Additional file 1). Results from single PCRs performed on these 225 strains to amplify distinct genomic regions located along the entire *fhlA-nlpD* region were consistent with the gene patterns and sizes deduced from the control strains (Table 1). RFLP type A (n = 69 strains) was the most predominant restriction pattern and was exclusively constituted by phylogroup B2 and *o454-nlpD* pattern III strains of different multilocus sequence types, particularly of those belonging to the ST complex STC95 (88.4%). Another 37 strains were assigned to RFLP type B that resembles the CFT073 AluI-restriction profile. More than two thirds of the strains allocated to this RFLP type were assigned to five different sequence types of the B2-ST complex STC73, while single strains were also assigned to STs of other clonal complexes, including ST131, ST12, ST144, and ST355, all of which are allocated to the B2 group. Likewise, other RFLP types are linked with certain phylogenetic backgrounds such as type D with B2-ST141, type E with D-ST115 and D-ST68, type F with ABD-ST117, type H with D-ST38, type J with ABD-ST62, type P with B1-STC23 or type Q and type R with A-STC10. Only exceptionally, different phylogenetic groups are associated with one RFLP type, while the clustering of different STs of same phylogenetic groups within a single RFLP type was frequently observed.

### 2.2 Serogroups

Among the *E. coli* strains subjected to serotyping (n = 426) 62 different O-antigens and 29 different H-antigens were determined (see Additional file 2). The most commonly occurring serogroups were O1:H7 (n = 15), O78:NM (n = 14), O18:H7 (n = 16), O6:H1 (n = 13), and O2:H5 (n = 10). While O78:NM was associated almost exclusively with pattern II, all the others were strictly associated with pattern III with only one exception. A number of isolates revealed an O antigen which was either not typable (n = 74) or rough (n = 12). Another 45 isolates harboured a flagella antigen which could not be assigned a known type.

### 2.3 Association between o454-nlpD genomic region and sample category, MLST, phylogroup, class/pathotype, host species, clinical condition and serotype

**Figure 4:**
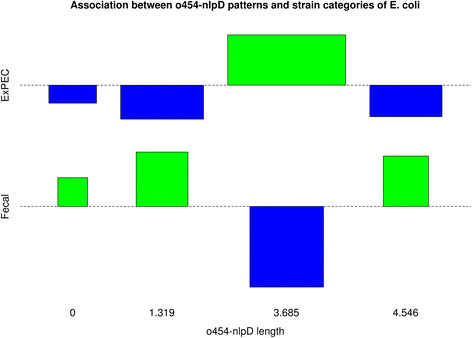
**Cohen-Friendly association plot showing the association between*o454-nlpD*patterns and strain categories (Fecal and ExPEC) of*E. coli*(n = 510).** The association was obtained calculating the Person’s Chi-squared test using the R software.

Person’s chi-squared test showed a strong association between the *o454-nlpD* patterns and the strain categories “Faecal/Commensal” and “ExPEC”, with a *p*-value < 0.01 (*p* = 0.008). In particular, the Cohen-Friendly association plot revealed a positive and exclusive association between the pattern III and the ExPEC category (Figure 4). Differently, the faecal category was positively associated to all other patterns.

Due to the high number of nominal variables, the strength of association between *o454-nlpD* patterns and one of the variables sequence type, phylogroup, class/pathotype, host species, clinical condition and serotype, was investigated using a Cramer’s V test (Table 3). The *o454-nlpD* regions and MLST groups were strongly associated with a *Cramer’s V* value of 0.99. The Theil’s symmetric Uncertainty Coefficient (uc) revealed that knowing the values of the independent MLST variable, the uncertainty about the values of the dependent variable *o454-nlpD* pattern decreased by about 98% with a significant *p*-value of zero (*p.uc.CR* = 0), but not *vice versa*. Similarly to the MLST, a very strong association between *o454-nlpD* regions and the phylogenetic group was found (*Cramer’s V* = 0.75). In this case, the uncertainty on predicting one of the variables from another on average was about 65% (*uc.sym* =0.65). Statistical analysis revealed a strong association (*Cramer’s V* = 0.76) between the *o454-nlpD* patterns and the serogroup. However, the *uc.sym* of 0.27 revealed a probability of only 27% on correctly predicting the two variables from each other. A weaker relationship was found between *o454-nlpD* regions and class/pathotype, host species, clinical category, and clinical condition.

**Table 3:** **Strength of association between the** ***o454-nlpD*** **regions and the MLST and Ecor groups, class or pathotype, host species, clinical category, and serogroup**

	***cramers.v***	***uc.RC***	***p.uc.RC***	***uc.CR***	***p.uc.CR***	***uc.sym***
**MLST**	0.99	0.28	0	0.99	0	0.44
**Ecor**	0.75	0.58	1.49e-228	0.72	4.63e-284	0.65
**Class/Pathotype**	0.22	0.05	2.79e-06	0.06	3.64e-06	0.05
**Host species**	0.31	0.08	1.42e-13	0.11	2.23e-11	0.09
**Clinical category**	0.19	0.03	1.38e-05	0.05	1.57e-05	0.04
**O-groups**	0.63	0.16	2.12e-65	0.45	1.8e-62	0.24
**H-groups**	0.56	0.17	1.48e-47	0.38	1.77e-42	0.24
**Serogroup**	0.84	0.19	8.93e-156	0.75	1.65e-272	0.3

The values *Cramer’s V*, *uc.RC*, *p.uc.RC*, *uc.CR*, *p.uc.CR* and *uc.sym* are reported here. *Cramers.v* measure the association between two nominal variables, giving a value between 0 and +1 (inclusive); *uc.RC* is the Theil’s symmetric Uncertainty Coefficient UC, indicating how much knowing the values of the Row variables decreases uncertainty about the Column variables; *p.uc.RC* is the probability of gaining UC(R|C) by chance; *uc.CR* is the Theil’s symmetric Uncertainty Coefficient UC, indicating how much knowing the values of the Column variables decreases uncertainty about the Row variables; *p.uc.CR* is the probability of gaining UC(C|R) by chance; *uc.sym* is a combination of *uc.RC* and *uc.CR*. The association was evaluated with the R-package “polytomous”. The two-way contingency tables used for this analysis are reported in Additional files (see Additional file 2, Additional file 5, Additional file 6, Additional file 7, Additional file 8 and Additional file 9).

### 2.4 Virulence associated genes and o454-nlpD genomic region

Among 47 VAGs tested the non-fimbrial adhesin gene *nfaE* was absent in all strains, while the other 46 VAGs occurred differently in our data set. For each VAG the significance of the pattern association in terms of *p*-values, the pattern to which they are associated with and the type of associations (positive/negative) were investigated and reported in Table 4. Statistical analysis revealed that pattern III, which generally harboured the highest mean number of VAGs (23.1 ± 4.6) compared with strains of patterns I (13.1 ± 5.0), II (10.7 ± 5.7), and IV (12.6 ± 6.2), was exclusively associated with 26 VAGs, 23 of which were positively, and three negatively associated. Pattern III was also significantly associated with another twelve VAGs, but not in an exclusive way. For instance, the curli gene *csgA* and the bifunctional adhesin and iron receptor gene *iha* were negatively associated with pattern III together with pattern I and II, respectively. Genes such as *hra* (heat-resistant agglutinin), *papC* (Pap-fimbrial subunit), *chuA* (haem utilization gene), *kpsMTII* (capsule group II synthesis), *sat* (secreted autotransporter toxin) and *tia* (toxigenic invasion locus A) were positively associated with pattern III together with pattern I, while the genes *ompT* (outer membrane protein), *traT* (transfer protein), *hlyF* (hemolysin) and *pic* (serin protease) were positively associated with pattern III together with pattern IV. No significant association between *o454-nlpD* regions and adhesion genes *afa/draBC*, *bmaE*, *gafD*, and *tsh*, iron acquisition genes *iucD*, *iutA*, and *sitD*_chromosomal_, and with serum resistance gene *ompA* was found.

**Table 4:** **Virulence-associated genes with significant association to** ***o454-nlpD*** **patterns**

**VAG**	***P*-value**	**Association with pattern**	**Type of association**
*focG*	1.18e-15	III	+
*papAH*	8.50e-25	III	+
*papEF*	3.83e-11	III	+
*papG II/III*	3.32e-20	III	+
*sfa/foc*	1.23e-33	III	+
*sfaS*	6.01e-15	III	+
*fyuA*	7.10e-25	III	+
*ireA*	1.99e-08	III	+
*iroN*	3.62e-40	III	+
*irp2*	7.14e-42	III	+
*sitD*_*chromosomal*_	6.87e-80	III	+
*colV*	0.000	III	+
*iss*	0.001	III	+
*neuC*	1.96e-28	III	+
*cnf*	1.30e-17	III	+
*vat*	2.41e-76	III	+
*hlyA*	1.39e-20	III	+
*ibeA*	3.96e-22	III	+
*gimB*	1.12e-35	III	+
*malX*	2.03e-94	III	+
*pks*	3.53e-49	III	+
*puvA*	6.94e-05	III	+
*ea/I*	1.59e-67	III	+
*fimC*	4.52e-06	III	-
*mat*	0.047	III	-
*astA*	0.002	III	-
*hrA*	3.39e-05	I, III	+
*papC*	2.31e-19	I, III	+
*chuA*	6.42e-78	I, III	+
*kpsMTII*	9.17e-42	I, III	+
*ompT*	0.001	III, IV	+
*traT*	0.034	III, IV	+
*sat*	0.007	I, III	+
*hlyF*	0.001	III, IV	+
*tia*	4.84e-05	I, III	+
*pic*	3.95e-14	III, IV	+
*csgA*	6.44e-85	I, III	-
*iha*	7.91e-05	II, III	-

*P*-values obtained with Fisher’s exact test and type of association with *Cohen-Friendly* association plot. No significant association was observed for genes *afa/draBC* (*p* = 0.463), *bmaE* (*p* = 0.085), *gafD* (*p* = 0.257), *tsh* (*p* = 0.386), *iucD* (*p* = 0.565), *iutA* (*p* = 0.506), *sitD*_episomal_ (*p* = 0.89), and *ompA* (*p* = 0.354).

### 2.5 Random forest and importance of VAGs for classification of E. coli population into patterns

**Figure 5:**
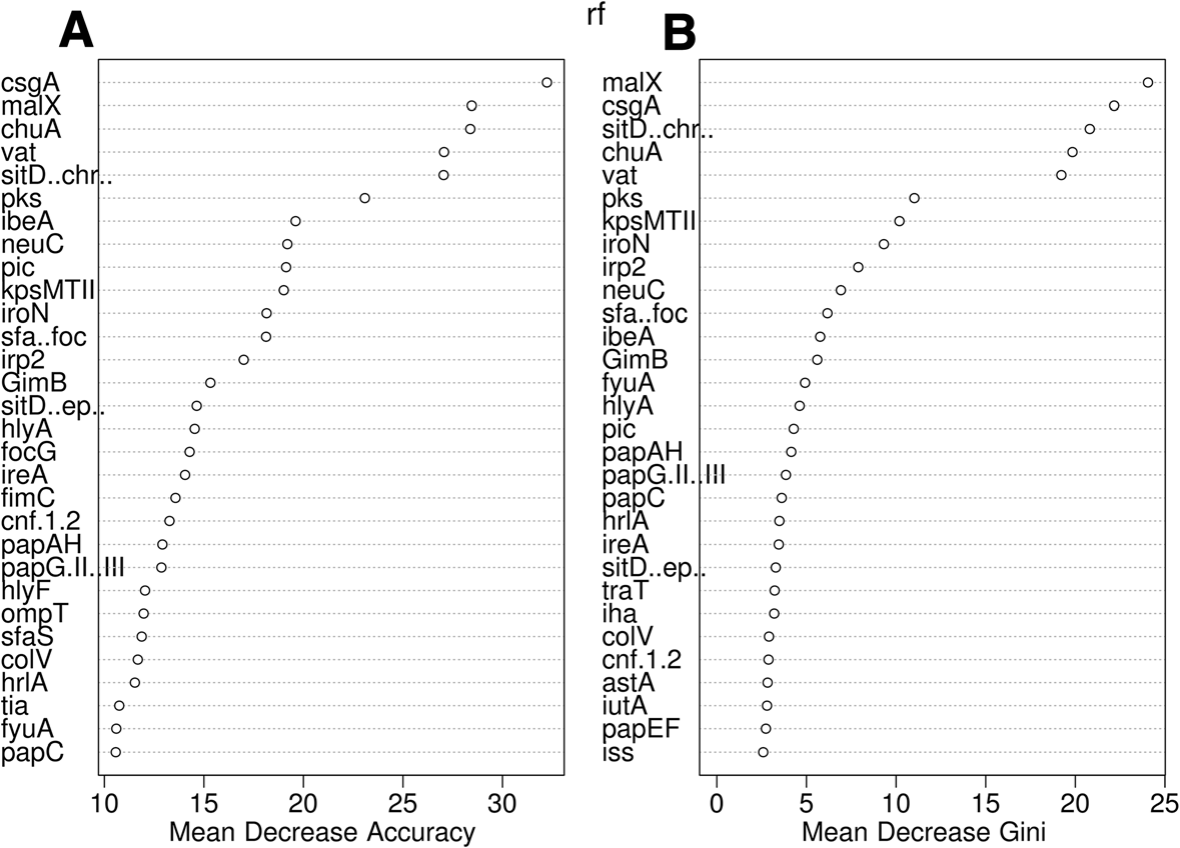
**VAGs importance for classification of the*o454-nlpD*patterns. A)** VAGs ordered according to the mean decrease accuracy score. Values calculated from the 70% of the data set; **B)** VAGs ordered according to the mean decrease Gini score. Value based on the OOB (out-of-bag) data. The analysis was performed with the R-package “Random Forest”.

In order to interpret the relevance of VAGs variables for pattern prediction of the data set under study and to filter out non-informative VAGs, Random Forest algorithm was used. The RF classification of the *o454-nlpD* patterns estimated an out-of-bag (OOB) error rate mean of 18.43% when using 46 VAGs. The OOB error rate for classification of each pattern is reported in Table 5. Only pattern III showed an error rate of zero, indicating a good performance of RF for pattern III prediction. The first most important genes for classification, as measured by the mean decrease in accuracy, were genes for curli adhesin CsgA, pathogenicity island marker MalX which represents a major carbohydrate active-transport system, haemin uptake system ChuA, vacuolating autotransporter toxin Vat, and iron and manganese transporter SitD (chromosomal gene variant) with values ranging between 25 and 30 (Figure 5a). Similar results were obtained measuring the importance of each VAG using the OOB data and the mean decrease Gini scores as shown in Figure 5b.

**Table 5:** **OOB (out-of-bag) error rate for** ***o454-nlpD*** pattern classification*

	**Pattern I**	**Pattern II**	**Pattern III**	**Pattern IV**	**Class. error**
**Pattern I**	29	0	1	11	0.29
**Pattern II**	0	115	5	4	0.07
**Pattern III**	0	0	251	0	0
**Pattern IV**	15	51	7	21	0.78

*o454-nlpD* patterns: I = *o454*-negative, II = 1.319 bp, III = 3.685 bp, IV = 4.546 bp.

*One *E. coli* strain which carries the *IS*2000 in the *o454-nlpD* region is not included here.

### 2.6 Phylogenetic analysis of the E. coli mutS gene

**Figure 6:**
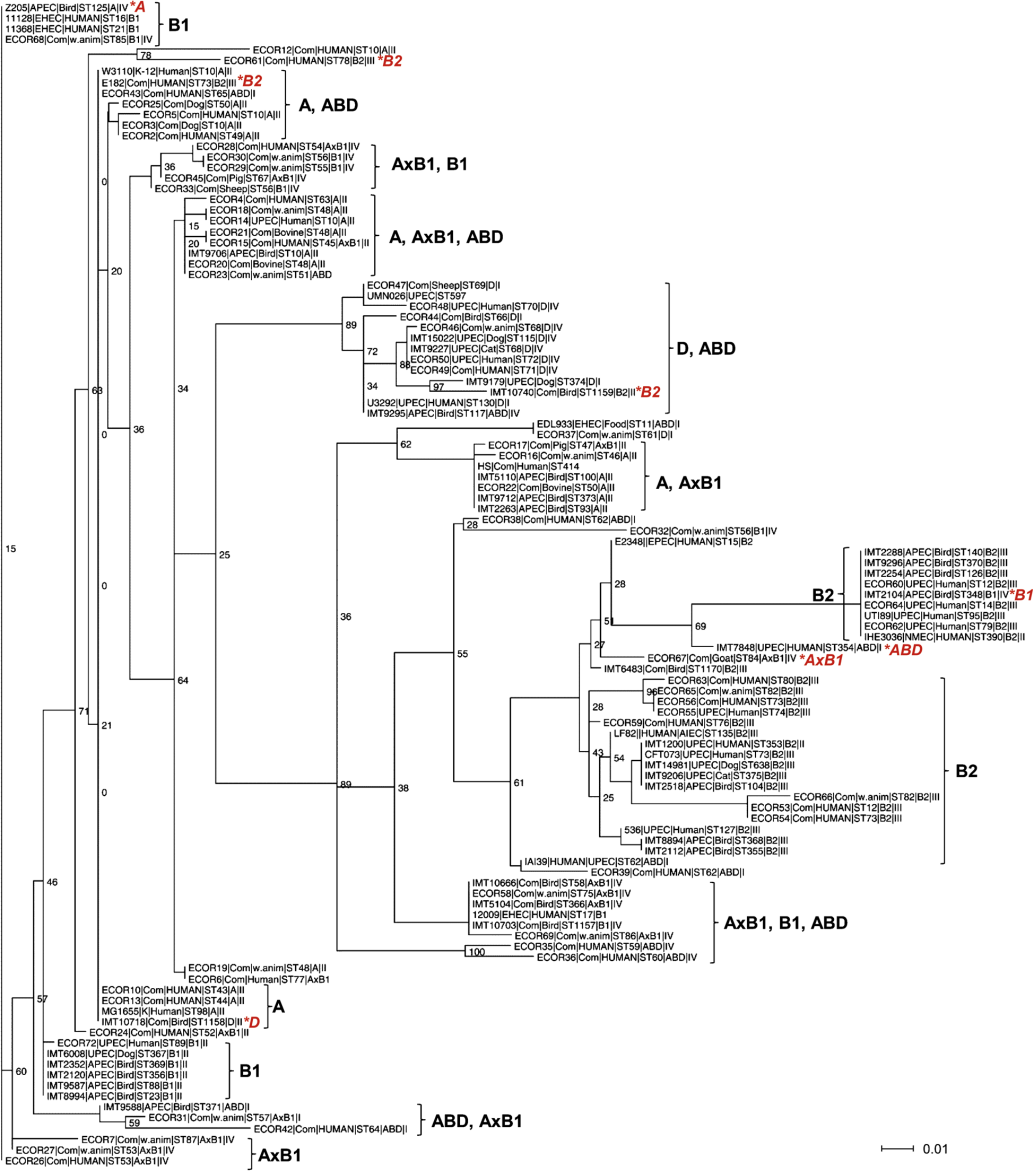
**Phylogenetic comparisons of*mutS*among*E. coli*strains of different pathotypes, host origin, sequence type (ST) and phylogenetic group.** The coloured letters indicate discordance between the clustering of *mutS* gene and the phylogenetic background of the strains. For a better overview, strains with identical *mutS* allele, ST, phylotype, and pathotype are not shown here. A full picture of the comparison of *mutS* and concatenated MLST allele sequences which contains all 177 strains is provided as additional file (Additional file 3).

Comparison of 177 partial *mutS* gene sequences generated in this study (n = 85 strains representing different *o454-nlpD* patterns, pathotypes, hosts, and multilocus sequence types), described in a previous study [14] (n = 72 Ecor strains; n = 20 *mutS* sequences obtained from available whole genome sequences), and obtained from 20 whole genome sequences of various *E. coli* pathotypes revealed 60 different *mutS* alleles. Although some of the bootstrap values show a low support for certain branches of the tree for the *mutS* sequence dataset a significant clustering of the *mutS* alleles can be observed (Figure 6). Many of these clusters are according to the population structure given from the housekeeping genes phylogeny (see Additional file 3), as deduced from the MLST dataset and corresponding assignment to Ecor groups performed by structure analysis. Yet there are some strains that contain a *mutS* from a different phylogroup. For example, in APEC strain IMT2104 and avian commensal strain IMT10740 the Ecor group B2 is involved which is usually a very distinct phylogenetic group. These observations confirm former analyses about the differences in the evolution of *mutS* compared to the core genome. One possible explanation for this phenomenon could be a different recombination rate for the region containing the *mutS* gene.

## 3 Discussion

The *mutS-nlpD* region represents a major operational region of genomic evolution in Enterobacteriaceae. Previous studies suggested that the polymorphic nature of this genetic region due to high mutation rate and to loss or acquisition of genes by horizontal gene transfer plays an important role in the constant adaptation of the bacteria to environmental changes and to new ecological niches [2],[14]–[16]. So far, the mosaic structure of the *mutS-nlpD* region was mainly investigated in intestinal pathogenic strains. By that, RFLP type analysis of the *fhlA-nlpD* genomic region revealed four different clusters: i.e. K-12 and Ecor group A, EPEC 1 group, EPEC 2 & EHEC 2 groups, and O157:H7 & EHEC 1 group [16]. Soon after, Le Clerc et al. [19] detected a polymorphism at the proximity of the *rpoS* gene. In particular, a 2.9-kb DNA insertion was identified in *E. coli* O157:H7 and related enterohaemorrhagic *E. coli* (EHEC) strains as well as in *Shigella dysenteriae*[19], while a 2.1-kb DNA insert was observed in *E. coli* pyelonephritis strain CFT073 [15]. These authors investigated diverse clinical isolates, including 23 from urinary tract infections (UTIs), 26 from infantile diarrhoea and haemorrhagic colitis, and seven from catheter-associated infections. Since the DNA insert previously identified in UPEC strain CFT073 was more common among urinary tract infection isolates (82.6%) than among other clinical *E. coli* isolates they suggested it to be specifically linked with uropathogens [15]. They also stated, that all B2 strains of the *E. coli* reference collection harboured this specific DNA insert und concluded that it might also predict the virulence of a strain, as B2 strains are known to be highly virulent in terms of extraintestinal pathogenicity.

From these studies it seemed likely that there might be pathotype- or virulence-associated polymorphisms in the *mutS-rpoS* region of the *E. coli* chromosome, although this has not been verified by using a large set of field strains. In the present study, PCR analyses of the *o454-nlpD* intergenic sequence from 510 predominantly extraintestinal pathogenic and commensal *E. coli* strains revealed substantial size variation. With only few exceptions, the strains were grouped into four patterns which resembled previously identified groups determined for the O157:H7 & EHEC 1 group (termed as pattern I in our study), K-12 and Ecor A group (pattern II), CFT073 and other uropathogens (pattern III), and the EPEC group (pattern IV) [15]. While patterns I, II, and IV were randomly distributed among all non-B2 group strains, pattern III was exclusively observed in group B2 strains, both of clinical and faecal origin. This is consistent with the genomic variation in selected Ecor strains described by others [15],[19],[23],[24]. With respect to the ongoing evolution and due to observations from previous MLST analyses [8] it is generally recognized that the Ecor collection does not reflect the entire *E. coli* population. Previous findings about the presence of the *rpoS*-proximal 2.1-kb insertion in all Ecor group B2 isolates and in a limited number of clinical isolates could thus not be extrapolated to nowadays relevant ExPEC-sequence types, such as ST95, ST127, ST372, and the global emerging multiresistant ST131 clone [25]–[29]. In our strain collection, these sequence types as well as other clinically important B2-STs, including ST73 and ST80 [28],[30] were frequently represented suggesting the existence of a B2-associated *mutS-nlpD* intergenic region.

Since pattern III was found to be highly associated to the strain category ExPEC and with a higher number of ExPEC-related VAGs we could speculate a potential role of these genes with the ability of the strain to invade extraintestinal tissues. However, replacement of *slyA* with other genes such as *o347* and *o183* in pattern III and their roles in pathogenesis remain to be investigated. *SlyA*, found exclusively in the EHEC- and EPEC-related patterns I and IV plays an important role in the invasion and survival of *Salmonella* in macrophage cells [31]. The relevance of this transcriptional regulator for the pathogenesis of non-B2 ExPEC strains has not been explored yet. Similarly, the putative role of the *o347*-encoded factor, showing a limited level of similarity to enzymes implicated in antibiotic hydrolysis, remains to be determined [15],[32]. Girardeau et al. [33] investigated common traits between animal and human ExPEC isolates positive for afimbrial adhesin gene *afa-8.* Interestingly, they found a significant proportion of human pyelonephritis-associated isolates showing the *mutS-rpoS* intergenic region found in EPEC and EHEC isolates. Furthermore, a putative function of *SlyA* in the pathogenesis of certain extraintestinal *E. coli* isolates which largely lack ExPEC-associated traits, such as S-fimbrial gene *sfa*, alpha-hemolysin gene *hly*, cytotoxic necrotizing factor gene *cnf*, was suggested. In accordance with our data, Girardeau et al. [33] and others observed *mutS-rpoS* intergenic patterns among ExPEC strains, which were previously associated with intestinal pathotypes [15],[33]. Thus, with respect to the group of ExPEC and its various pathotypes this genetic region may not be regarded as pathotype-associated but more likely as a marker to reflect their diversity and probably to predict highly virulent members of this group, as different patterns are obviously linked with single ExPEC-related virulence genes.

Advance statistics like Random Forest (RF) algorithm was used to resolve difficult tasks such as the identification of the most important VAGs for the prediction of the *o454*-*nlpD* patterns. The RF classification of the *o454*-*nlpD* patterns estimated an OOB (out-of-bag) error rate of 18.4% when using 46 virulence-associated genes (VAGs). This relatively high error rate clearly indicated that not all VAGs used were strongly associated with a specific *o454*-*nlpD* pattern as shown in Table 3 where only pattern III is associated to specific genes. Other genes where associated to at least two different patterns. However, since the error rate in predicting the pattern III was zero (Table 5 and Additional file 4) we can conclude that RF performed very well for pattern III prediction. Furthermore, RF allowed us to identify for the first time the top-ranked VAGs for pattern III prediction. The most predictive indicators, the genes *csgA*, *malX*, *chuA*, *vat* and *sitD*_chromosomal_ were also statistically significant predictors, and thus worthy of further investigation. Since pattern III was nearly exclusively linked with group B2 strains in our study, these genes may also be regarded as predictive for highly virulent members of the ExPEC group and of commensal *E. coli* harbouring virulence potential, respectively. Indeed, the heme binding protein encoding gene *chuA* is one of the genetic regions targeted in the PCR-based approach for rapid phylogenetic typing of *E. coli* strains and is said to occur regularly in group B2 and D strains [9],[34]. *ChuA*, together with *vat*, which encodes an autotransporter serine protease toxin, *fyuA*, which encodes the yersiniabactin receptor and finally a gene (*yfcV*), which encodes the major subunit of a putative chaperone-usher fimbria, have previously been included in a diagnostic multiplex PCR to identify strains of the UPEC pathotype [35]. In their study, Spurbeck et al. [35] suggested that *E. coli* isolates that encode these four genes are correlated with high numbers of other VAGs, are able to colonize the bladder in higher numbers than strains lacking these genes, and are nearly 10 times more likely to represent UPEC or NMEC strains than faecal commensal strains [35]. Likewise positively linked with pattern III strains is *malX*, which codes for a phosphotransferase system enzyme II that recognizes maltose and glucose [36] and is frequently present in ExPEC strains [37]–[39]. Östblom et al. [40] could demonstrate, that *malX* was among those genes that were associated with fitness of *E. coli* in the infant bowel microbiota. Here, carriage of various pathogenicity island markers and particularly *malX* correlated positively with the time of persistence of individual strains in the colon, supporting their role to increase the fitness of *E. coli* in its natural niche, the colon [38].

The *mutS* chromosomal region has long been identified as the location for the insertion of blocks of VAGs e.g. in the case of the two widely diverged pathogens, *Salmonella* Typhimurium and *Haemophilus influenzae*. In *S.* Typhimurium, a 40 kb pathogenicity island (SPI-1) is inserted 5′ to the *mutS* gene [40] and in *H. influenzae*, a 3.1 kb tryptophanase gene cluster (*tna*) is inserted on the 3′ side of the *mutS* gene in strains that cause spinal meningitis in infants [41]. This insertion allows the utilization of tryptophan and, thus, provides a growth advantage for the pathogen, particularly in the tryptophan-rich environment of cerebrospinal fluid. In our study, there was no indication for an insertion of PAI-like structures or of larger blocks of VAGs in the intergenic *mutS-rpoS* region in any of the 510 *E. coli* strains under investigation. Other studies raised the hypothesis that a certain *o454-nlpD* pattern, reflecting distinct evolutionary *E. coli* lineages, might be linked with the acquisition of VAGs at chromosomal sites outside this genetic region by HGT [2],[15],[42]. Culham and Wood [15] suggested that the 2.1-kb insertion upstream of *rpoS* arrived earlier than certain virulence determinants linked with urinary tract infections, such as genes for P-fimbriae (*pap*), S-fimbriae (*sfa*), a polyketide synthetase (*pks*), and α-hemolysin (*hly*) during the evolution of group B2. Basically, the polymorphism in this genetic region is considered to result from the close linkage of *mutS* and *rpoS* genes which are frequently mutated in *E. coli* evolution due to ecological specialization upon repeated shuttles between different environments [2]. Here, their inactivation as well as the re-acquisition of functional alleles might have been of selective advantage, e.g. in terms of stress resistance, higher mutation rates, genome plasticity, and stabilization of beneficial adaptive mutations [5],[43].

Indeed, in addition to the findings of genetic variability, phylogenetic analysis of EHEC and EPEC pathogens, as well as strains of the Ecor collection, revealed that an unexpected level of recombination between *mutS* genes has occurred during the evolution of these strains [14],[20]. In a comparison of *mutS* phylogeny against predicted *E. coli* “whole-chromosome” phylogenies, derived from multilocus enzyme electrophoresis (MLEE) and *mdh* sequences, Brown et al. [14] observed striking levels of phylogenetic discordance among *mutS* alleles and their host strains, which basically represented the Ecor collection. To investigate whether this is also true for a greater collection of strains and using concatenated MLST gene sequences instead of single genes or MLEE as comparison, we extended this approach on 177 ExPEC and commensal strains. Here, many of the *mutS* alleles clustered according to the population structure given from the housekeeping genes phylogeny, indicating a low frequency of recombination events across phylogenetic groups. However, for a number of strains we also found incongruence between these two phylogenies, which is likely due to recombination of *mutS* between different phylogenetic groups. By investigating the molecular phylogeny of MMR (methyl-directed mismatch repair) genes from natural *E. coli* isolates Denamur et al. [20] could show that, compared to two housekeeping genes, individual functional MMR genes exhibit high sequence mosaicism derived from diverse phylogenetic lineages. They suggested that the MMR functions have frequently been lost and reacquired in the evolution of *E. coli*. To which extent *mutS* and other genes of the MMR system as well as the *mutS-nlpD* intergenic region represent a hallmark of a mechanism of adaptive evolution in ExPEC and in other *E. coli* pathotypes has been scarcely investigated. Certainly, *mutS* has a unique role in the formation of mutators with relaxed recombination barriers, and bacteria with a defect in their MMR system, e.g. by a temporary loss of *mutS*, are more prone to genetic variations and HGT and, consequently, have an increased capacity to adapt to the host environment or acquire new VAGs, respectively [2],[44]. This phenomenon was recently described for UPEC strains, where *mutS* and other genes of the MMR system were found to be involved in the reciprocal control of motility and adherence of UPEC due to an increased expression of flagellin [44]. The authors discussed a possible relationship between MutS and UPEC pathogenesis in general as urinary tract isolates exhibit a higher occurrence of mutator strains than commensal *E. coli* or any other *E. coli* pathotype [42].

## 4 Conclusions

The *mutS-rpoS* intergenic region of ExPEC and commensal *E. coli* strains resembles a great phylogenetic diversity of these strains as exemplified by the presence of four different patterns. The grouping based on *o454-nlpD* amplicons and RFLP patterns of the entire *mutS-rpoS* region was in accordance with other genotype-based methods, like MLST and Ecor typing. Significant associations were determined between pattern III and phylogenetic group B2 strains, representing the most virulent members of the ExPEC group. *MutS* alleles revealed an evolution parallel to their phylogenetic background with few exceptions. The random forest algorithm allowed for the first time the identification of the most important virulence genes for prediction of the most virulent ExPEC strains.

Our results suggest the *mutS-rpoS* region to be of great value in identifying highly extraintestinal virulent strains among the mixed population of *E. coli* promising to be the basis of a typing tool for ExPEC and their reservoir.

## 5 Methods

### 5.1 Bacterial strains

A total of 510 *E. coli* isolates, including 72 strains of the *E. coli* reference collection (Ecor) were examined for their *o454-nlpD* genomic region. This strain collection comprised of 367 extraintestinal pathogenic *E. coli* (ExPEC) isolated from multiple anatomical sites and 143 strains isolated from the faeces of clinically healthy humans (n = 83) and animals (n = 60). Pathogenic strains were isolated from urinary tract infections in humans (n = 65) and animals, including dogs, cats, horses, and pigs (n = 93), avian systemic and local *E. coli* infections (collectively termed colibacillosis) (n = 135), meningitis in infants (n = 24), septicaemia in humans (n = 28) and animals (n = 5), and from other diseases, such as peritonitis, mastitis, metritis, cervicitis, and vaginitis in various animal species (n = 18), the latter three diseases broadly categorized as genital tract infection. Clinical strains were recovered during routine microbiological diagnostic of samples from patients in hospitals and veterinary clinics in Germany. In case of disease outbreaks, only one strain per event was included. *E. coli* isolated from healthy individuals were avian faecal strains, which originated from cloacal swabs of clinically healthy poultry from Germany and human commensal strains isolated from the gut of healthy human carriers published earlier [45]–[47]. Commensal strains belonging to the Ecor collection originated from the gut of clinically healthy animals, such as cattle, dogs, sheep, pigs, and orang-utan [48].

We further included enteropathogenic *E. coli* E3248/69 [49] and enterohaemorrhagic *E. coli* EDL933 [50] as well as K-12 laboratory strain MG1655 as references for PCR mapping of the *mutS-nlpD* genomic region. Strains were stored at −70°C in brain heart infusion broth with 10% glycerol until further use.

### 5.2 DNA preparations

Bacterial DNA was extracted using the Master-Pure™ Genomic DNA Purification Kit (Biozym Diagnostik GmbH, Hessisch Oldendorf, Germany) according to the manufacturer’s recommendations. DNA concentrations were determined by NanoDrop ND-1000 spectrophotometer. The DNA was diluted in MilliQ sterilized water to obtain ca. 50 ng/μl, and 4 μl were used for single and multiplex PCR.

### 5.3 Characterization of the o454-nlpD genomic region and sequence analysis of mutS

In order to investigate the *o454-nlpD* region size a PCR approach was used. The amplification of the *o454-nlpD* regions was carried out with oligonucleotide primers F5 and R2 (Table 1) using standard PCR conditions. The reference sequences selected for were: MG1655 (U00096), EDL933 (NC_002655), CFT073 (NC_004431) and E2348/69 (FM180568). The structure of these genomic regions and the relative size of the reference sequences are illustrated in Figure 1.

Long range PCRs for amplification of the whole *fhlA*-*mutS* region, using primers *fhlA* FP and *nlpD* RP, were performed by an Extensor Hi-Fidelity PCR Master Mix (ABgene®, Fisher Scientific - Germany GmbH) as recommended by the manufacturer. Briefly, for a 25 μl reaction 10.0 μl of Extensor Master Mix was mixed with 0.2 μl of oligonucleotide primers in a 100 pmol concentration, 4 μl (400 ng) template DNA and 10.8 μl deionized water. The amplification was performed in a thermal cycler (Perkin Elmer GeneAmp® PCR System 2400, Applied Biosystems, Darmstadt, Germany) using the following program: 92°, 2 min for initial denaturation; 10 cycles of 92°C, 10 sec denaturation, 59°C, 30 sec annealing and 68°C, 8 min elongation; the following 15 cycles started with an extension time of 68°C, which was prolonged for 10 sec per cycle. A final extension cycle was applied at 68°C for 7 min.

*E. coli* strains were further investigated for various regions within the *fhlA*-*mutS*-*rpoS-nlpD* genomic region by PCR assays according to standard protocols [51]. Targeted genes, their descriptions as well as sequences of oligonucleotide primers and their positions within the target sequences are given in Table 1 and Figure 1.

Oligonucleotide primers used to amplify the *mutS* genes in *E. coli* were F1 and R3 and the sequences are given in Table 1. Amplification of *mutS* sequences was performed under the following conditions: initial denaturation at 94°C for 4 min; 30 cycles of 94°C for 45 sec, 58°C for 1 min, and 72°C for 2.30 min; and final incubation at 72°C for 10 min. After double strand sequencing of the amplicon (LGC Genomics, Berlin, Germany) the 380 bp-segment of the *mutS* gene, which corresponds to the conserved ATP-binding domain and to base pair coordinates 1808 to 2187 of the *mutS* coding region in *E. coli* CFT073 (GenBank accession number AE014075) was used for comparative phylogenetic analyses.

In addition to the 88 *mutS* alleles sequenced here, another 92 partial *mutS* sequences, originating from the Ecor strain collection (accession no. AF001987 - AF002010, AJ005826 - AJ005828, AF004287, AJ242620, and AF291185 - AF291258) and from fully sequenced strains CFT073 (UPEC, accession no. AE0140075), APEC_O1 (APEC, CP000468), UTI89 (UPEC, CP000243), HS (Commensal, CP000802), MG1655 (K-12, U00096), W3110 (K-12, AP009048), SMS-3-5 (Environmental, CP000970), 042 (EAEC, FN554766), 11128 (EHEC, AP010960), 11368 (EHEC, AP010953), 12009 (EHEC, AP010958), E2348/69 (EPEC, FM180568), EC4115 (EHEC, CP001164), EDL933 (EHEC, AE005174), H10407 (ETEC, FN649414), LF82 (AIEC, CU651637), O157:H7 Sakai (EHEC, BA000007), 536 (UPEC, CP000247), IAI39 (UPEC, CU928164), and UMN026 (UPEC, CU928163) were obtained from GenBank.

Phylogenetic trees of *mutS* sequences and concatenated sequences of the seven housekeeping genes included in MLST analyses were calculated using RAxML 8 [52]. For each phylogeny, 100 bootstrap replicates were calculated. The visualization of the tree was performed with Dendroscope 3 [53].

### 5.4 Nucleotide sequence accession numbers

The *mutS* nucleotide sequence data reported in this paper has been deposited in the GenBank sequence database with accession numbers KM232523 through KM232607.

### 5.5 Serotyping

Serotyping was performed on 426 strains at the Robert-Koch Institute (Wernigerode, Germany) by tube agglutination with rabbit anti-*E. coli* immune sera produced against a panel of antigenic test strains containing *E. coli* O-groups 1 to 181. Similar analysis was carried out in order to investigate the bacterial flagella antigens H group.

### 5.6 Multilocus sequence typing (MLST), Ecor grouping and phylogenetic analyses

Multi locus sequence typing was performed using the scheme published by Wirth et al. [8]. Allele sequences were allocated to the public database available at the MLST website (http://mlst.warwick.ac.uk/mlst/dbs/Ecoli). Ancestral groups were determined by an analysis based on the concatenated sequences of the seven housekeeping genes used for MLST. The linkage model implemented in the software Structure (http://pritch.bsd.uchicago.edu/software.html) was used to identify groups with distinct allele frequencies. Cut-off values for the assignment of individual isolates to one of the four groups (A, B1, B2, and D) as well as to hybrid groups AxB1 and ABD were determined according to Wirth et al. [8]. Phylogenetic clustering was performed by calculating a minimum spanning tree by means of a graphical software tool implemented in BioNumerics 7.1 (Applied Maths, Belgium). MLST data were partially adopted from previous publications [37],[45]–[47].

### 5.7 Virulence gene typing

Virulence associated genes (VAG)s of recognized importance in the pathogenesis of ExPEC strains were investigated by multiplex and single PCRs as described previously [37],[47]. VAGs investigated in the present study were 47 and encoded for factors within the categories of adhesins (*afa/draB*, *bmaE, csgA*, *fimC*, *focG, gafD, hrlA*, *iha*, *mat*, *papAH, papC*, *papEF, papG, sfa/foc*, *sfaS, tsh*), iron acquisition (*chuA*, *fyuA*, *ireA*, *iroN*, *irp2*, *iucD*, *iutA, sit*_episomal_, *sit*_chromosomal_), serum resistance/protectins (*iss*, *kpsMTII*, *neuC*, *ompA*, *ompT*, *traT*), toxins/hemolysins (*astA*, *cnf*, *sat*, *vat*, *hlyA*, *hlyF*), and invasion (*ibeA*, *gimB*, *tia*). Miscellaneous genes such as *malX*, *pic*, *puvA*, *pks*, and ColV plasmid operon genes *cvi/cva* were investigated as well.

### 5.8 Biostatistics

To analyse the significant relationship between two categorical variables with few nominal values, a Person’s chi-square test [54] was used. In case the assumptions of the chi-square test did not hold, a Fisher’s exact test was applied [55]. In particular, Person’s chi-square test was used to analyse the significant relationship between the *o454-nlpD* regions and the strain category (fecal or ExPEC), while Fisher’s exact test was employed to investigate the relationship between *o454-nlpD* patterns and single VAG. Cohen-Friendly association plot was used to visualize deviation from independence of rows and columns in a two-way frequency table [56]. *P*-values < 0.05 indicate that the relationship between the *o454-nlpD* regions and the strain category or single VAG is statistically significant. The Person’s chi-square test and Fisher’s exact test were performed using the functions “chisq.test” and “fisher.test” in R software (http://www.r-project.org).

To determine the strength of association between two categorical variables with multiple nominal values, a Cramer’s V test [57] was used. In particular, this type of statistic was applied to measure the association between the *o545-nlpD* patterns and one of the following variables: MLST type, Ecor group, class or pathotype, host species, clinical conditions and serotype. The two-way contingency tables fitted for Cramer’s V test (see Additional file 2, Additional file 5, Additional file 6, Additional file 7, Additional file 8 and Additional file 9) displayed as column names the *o454-nlpD* patterns and as row names one of the variables described above. *Cramers.v* values closer to 1 indicate a strong or high association between the two nominal variables, while closer to 0 indicate a weak or low association between them; *uc.RC* values, representing the Theil’s symmetric Uncertainty Coefficient UC, indicate how much knowing the values of the Row variables decreases uncertainty about the Column variables; *p.uc.RC* values indicate the probability of gaining UC(R|C) by chance; *uc.CR* values indicate how much knowing the values of the Column variables decreases uncertainty about the Row variables; *p.uc.CR* is the probability of gaining UC(C|R) by chance; *uc.sym* values closer to 1 indicate a lower uncertainty in predicting one of the variables from another on average. This analysis was performed using the R-package “polytomous” (http://cran.r-project.org/web/packages/polytomous/index.html).

In order to interpret the relevance of VAGs variables for pattern prediction and to filter out non-informative VAGs, Random Forest (RF) algorithm [21] was used. RF was performed using the R-packages “Random Forest” (http://cran.r-project.org/web/packages/randomForest/index.html) with the following settings: 1000 number of trees and 3 variables tried at each split. The result from RF and conditional variable importance was verified via multiple random forest runs starting with different seeds and sufficiently large number of tree values to ensure robustness and stability of results [58]. The commonly used importance measure from RF is the mean decrease Gini values. Gini value is directly derived from the “Gini index” on the resulting RF trees. The RF classifier uses a splitting function called “Gini index” to determine which attribute to split on during the tree learning phase. The Gini index measure the level of impurity of the samples assigned to a node based on a split at its parent.

### 5.9 Supporting data

The data sets supporting the results of this article are included within the article (and its additional files).

## Competing interests

The authors declare that they have no competing interests.

## Authors’ contributions

CE designed the experiments. CE, ID, HD, AF, and NN performed the experiments and contributed to acquisition and interpretation of data. CE and FD structured and prepared the manuscript. FD, TS and CE performed biostatistics. LHW and TS revised the manuscript critically for important intellectual content. All authors read and approved the final manuscript.

## Additional files

## Supplementary Material

Additional file 1: **Dendrogram based on restriction fragments of the *E. coli fhlA-nlpD*long PCR products digested with AluI.** RFLP patterns (A-S) were assigned based on identical restriction profiles of *E. coli* strains (at least two strains within a pattern). Strains revealing unique patterns were termed singletons. Among a subset of 225 *E. coli* strains included in the present study different RFLP patterns were represented by 69 strains (RFLP type A), B (n = 37), C (n = 3), D (n = 11), E (n = 3), F (n = 5), G (n = 5), H(n = 5), I (only EHEC reference strains), J (n = 5), K (n = 6), L (n = 3), M (n = 3), N (n = 5), O (n = 2), P (n = 15), Q (n = 17), R (n = 19), (n = 7), singletons (n = 8). Click here for file

Additional file 2: **Contingency tables showing the serogroup frequencies given the occurrence of the*o454-nlpD*patterns.** Table captions: *o454-nlpD* patterns: I = *o454*-negative, II = 1.319 bp, III = 3.685 bp, IV = 4.546 bp. Ont: O antigen not typable, although tested; Hnt: H antigen not typable, although tested; NM: non-motile. The most common serogroups are reported with bold character. Click here for file

Additional file 3: **Phylogenetic comparisons of concatenated MLST housekeeping gene alleles (*adk,fumC,icd,recA,purA,gyrB,*and *recA*) and *mutS*among 177*E. coli*strains of different pathotypes, host origin, sequence type (ST) and phylogenetic group.** Phylogenetic trees were calculated using RAxML 8 (open access link: http://bioinformatics.oxfordjournals.org/content/early/2014/01/21/bioinformatics.btu033.abstract?keytype=ref&ijkey=VTEqgUJYCDcf0kP). For each phylogeny, 100 bootstrap replicates were calculated. The visualization of the tree was performed with Dendroscope 3 (http://dendroscope.org). Click here for file

Additional file 4: **Plot of the OOB error rate per each*o454-nlpD*pattern.** The analysis was performed with the R-package “Random Forest”. Black line represents the error rate mean. Click here for file

Additional file 5: **Contingency tables showing the MLST frequencies given the occurrence of the*o454-nlpD*pattern.** The most common (n >10) sequence types (STs) are reported with bold characters. Table captions: *o454-nlpD* patterns: I = *o454*-negative, II = 1.319 bp, III = 3.685 bp, IV = 4.546 bp. Click here for file

Additional file 6: **Contingency tables showing the Ecor frequencies given the occurrence of the*o454-nlpD*pattern.** Table captions: *o454-nlpD* patterns: I = *o454*-negative, II = 1.319 bp, III = 3.685 bp, IV = 4.546 bp. Click here for file

Additional file 7: **Contingency tables showing the Class/Pathotype frequencies given the occurrence of the*o454-nlpD*pattern.** Table captions: *o454-nlpD* patterns: I = *o454*-negative, II = 1.319 bp, III = 3.685 bp, IV = 4.546 bp. Click here for file

Additional file 8: **Contingency tables showing the host species frequencies given the occurrence of the*o454-nlpD*pattern.** Table captions: *o454-nlpD* patterns: I = *o454*-negative, II = 1.319 bp, III = 3.685 bp, IV = 4.546 bp. Click here for file

Additional file 9: **Contingency tables showing the clinical category frequencies given the occurrence of the*o454-nlpD*pattern.** Table captions: *o454-nlpD* patterns: I = *o454*-negative, II = 1.319 bp, III = 3.685 bp, IV = 4.546 bp. Click here for file
