# Supplementary figures and images for: Correlation between the genomic o454-nlpD region polymorphisms, virulence gene equipment and phylogenetic group of extraintestinal Escherichia coli (ExPEC) enables pathotyping irrespective of host, disease and source of isolation
Source: Gut Pathog. 2014 Sep 16;6:37. doi: 10.1186/s13099-014-0037-x (PMC4209514; doi:10.1186/s13099-014-0037-x)

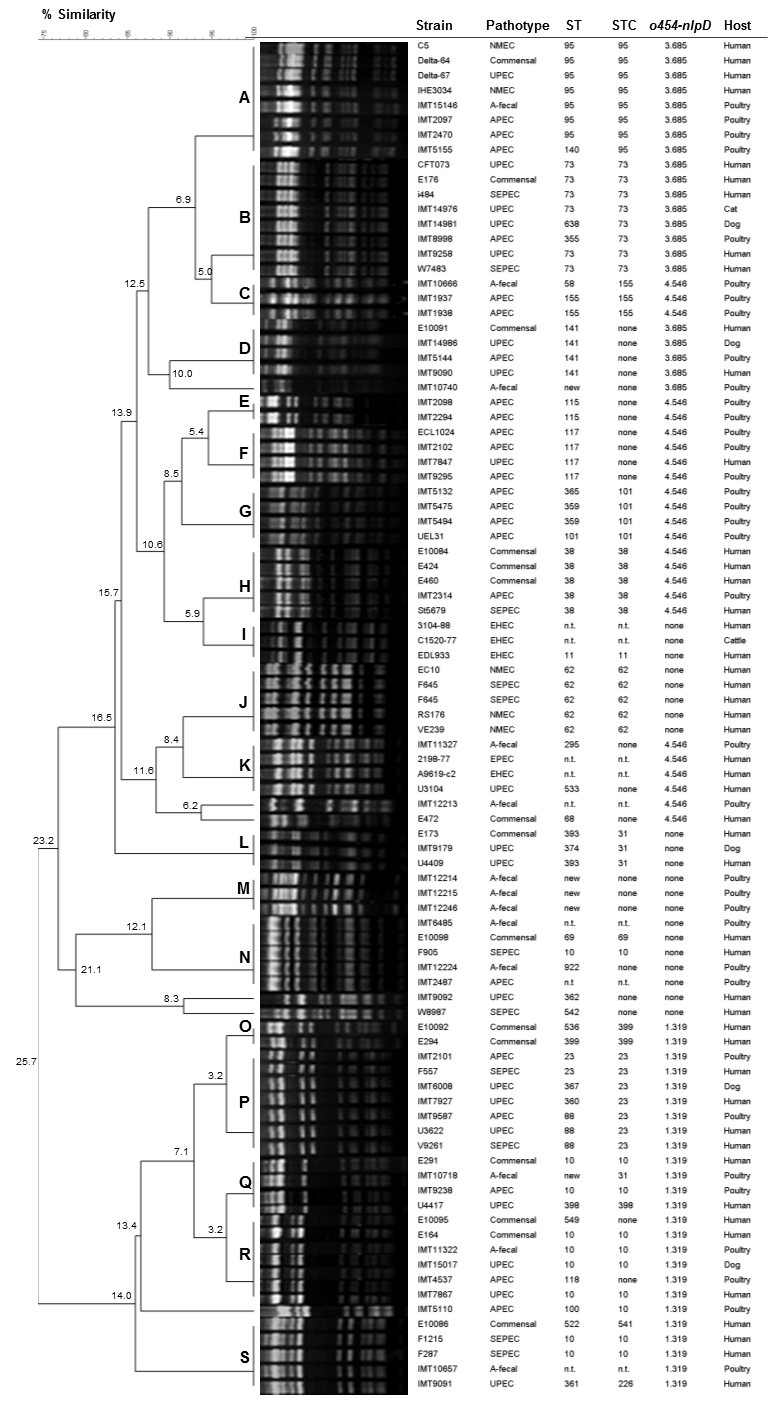

Supplement: Additional file 1: — Dendrogram based on restriction fragments of the E. coli fhlA-nlpDlong PCR products digested with AluI. RFLP patterns (A-S) were assigned based on identical restriction profiles of E. coli strains (at least two strains within a pattern). Strains revealing unique patterns were termed singletons. Among a subset of 225 E. coli strains included in the present study different RFLP patterns were represented by 69 strains (RFLP type A), B (n = 37), C (n = 3), D (n = 11), E (n = 3), F (n = 5), G (n = 5), H(n = 5), I (only EHEC reference strains), J (n = 5), K (n = 6), L (n = 3), M (n = 3), N (n = 5), O (n = 2), P (n = 15), Q (n = 17), R (n = 19), (n = 7), singletons (n = 8). [file s13099-014-0037-x-S1.png]

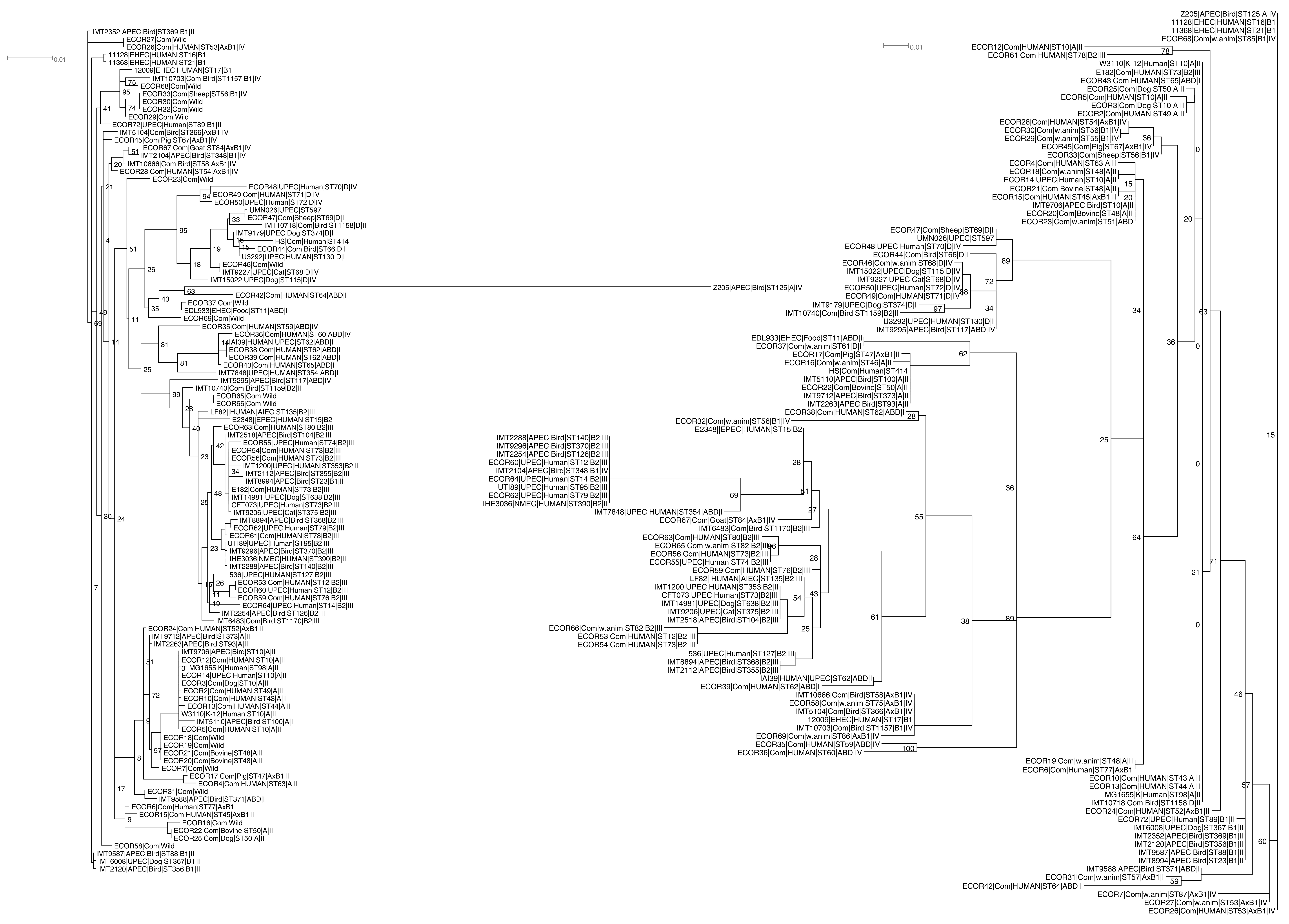

Supplement: Additional file 3: — Phylogenetic comparisons of concatenated MLST housekeeping gene alleles (adk,fumC,icd,recA,purA,gyrB,and recA) and mutSamong 177E. colistrains of different pathotypes, host origin, sequence type (ST) and phylogenetic group. Phylogenetic trees were calculated using RAxML 8 (open access link: http://bioinformatics.oxfordjournals.org/content/early/2014/01/21/bioinformatics.btu033.abstract?keytype=ref&ijkey=VTEqgUJYCDcf0kP). For each phylogeny, 100 bootstrap replicates were calculated. The visualization of the tree was performed with Dendroscope 3 (http://dendroscope.org). [file s13099-014-0037-x-S3.tiff]

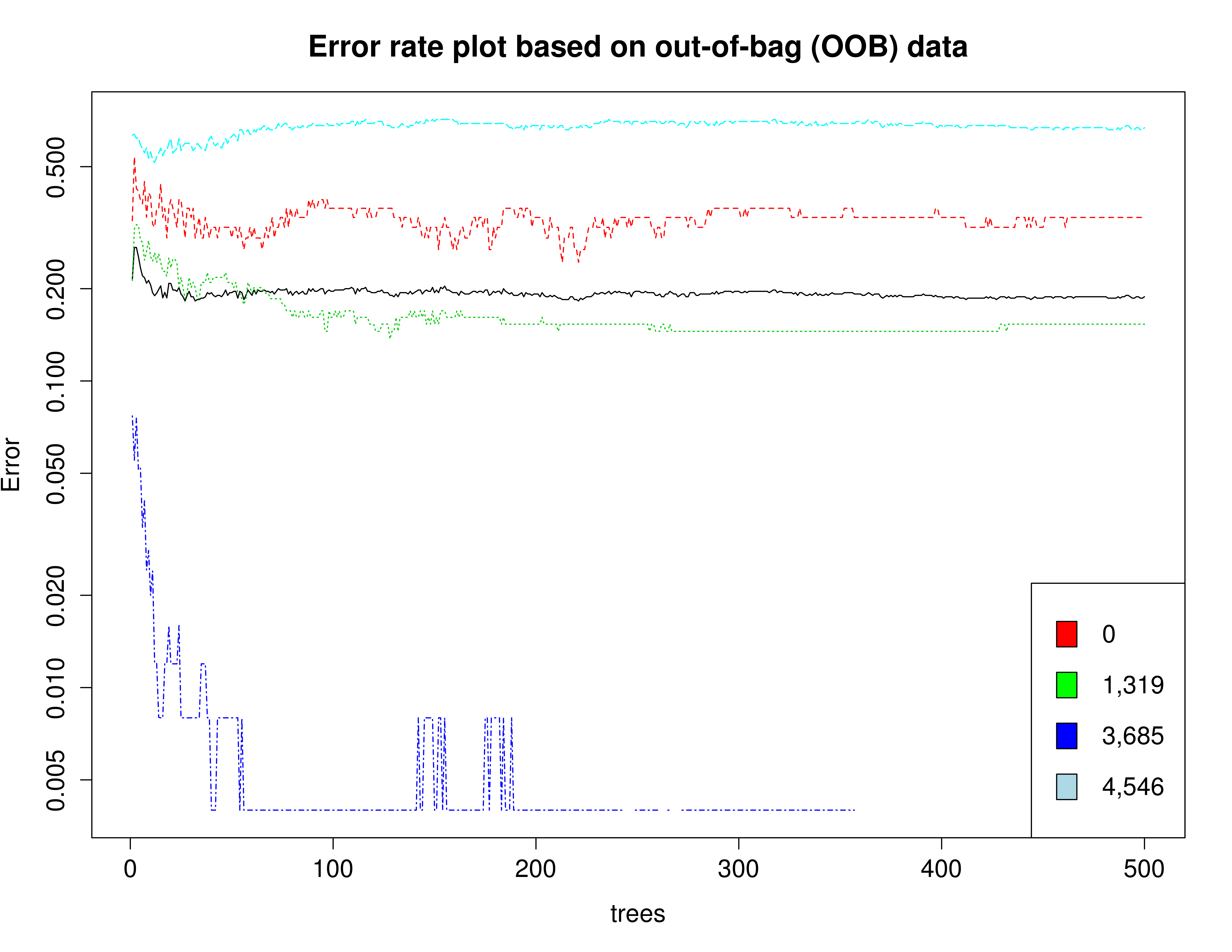

Supplement: Additional file 4: — Plot of the OOB error rate per eacho454-nlpDpattern. The analysis was performed with the R-package “Random Forest”. Black line represents the error rate mean. [file s13099-014-0037-x-S4.png]
