# Supplementary material for: Correlation between the genomic o454-nlpD region polymorphisms, virulence gene equipment and phylogenetic group of extraintestinal Escherichia coli (ExPEC) enables pathotyping irrespective of host, disease and source of isolation
Source: Gut Pathog. 2014 Sep 16;6:37. doi: 10.1186/s13099-014-0037-x (PMC4209514; doi:10.1186/s13099-014-0037-x)
Supplement: Additional file 2: — Contingency tables showing the serogroup frequencies given the occurrence of theo454-nlpDpatterns. Table captions: o454-nlpD patterns: I = o454-negative, II = 1.319 bp, III = 3.685 bp, IV = 4.546 bp. Ont: O antigen not typable, although tested; Hnt: H antigen not typable, although tested; NM: non-motile. The most common serogroups are reported with bold character. [file s13099-014-0037-x-S2.docx]

Additional File 2: Contingency tables showing the serogroup frequencies given the occurrence of the *o454-nlpD* patterns.

|  | ***o454-nlpD***  **pattern** | | | |  | ***o454-nlpD***  **pattern** | | | |  | ***o454-nlpD* pattern** | | | |  | ***o454-nlpD***  **pattern** | | | |
| --- | --- | --- | --- | --- | --- | --- | --- | --- | --- | --- | --- | --- | --- | --- | --- | --- | --- | --- | --- |
| **Serogroup** | **I** | **II** | **III** | **IV** | **Serogroup** | **I** | **II** | **III** | **IV** | **Serogroup** | **I** | **II** | **III** | **IV** | **Serogroup** | **I** | **II** | **III** | **IV** |
| not tested | 11 | 37 | 16 | 21 | **O18:H7** | 0 | 0 | 15 | 1 | O4:H31 | 0 | 0 | 1 | 0 | O86:Hnt | 0 | 1 | 0 | 0 |
| O1 | 1 | 0 | 4 | 2 | O18:NM | 0 | 0 | 4 | 0 | O4:H40 | 0 | 0 | 2 | 0 | O86:NM | 0 | 1 | 0 | 0 |
| O101:NM | 0 | 0 | 0 | 1 | O1:H15 | 0 | 0 | 0 | 1 | O4:H43 | 0 | 0 | 0 | 1 | O88:H5 | 0 | 0 | 1 | 0 |
| O102:NM | 0 | 0 | 0 | 1 | O1:H5 | 0 | 0 | 1 | 0 | O4:H5 | 0 | 0 | 1 | 0 | O88:H8 | 0 | 0 | 0 | 1 |
| O104:H21 | 0 | 0 | 0 | 1 | O1:H6 | 1 | 0 | 0 | 1 | O4:Hnt | 0 | 0 | 4 | 0 | O88:Hnt | 0 | 0 | 1 | 0 |
| O104:NM | 0 | 0 | 0 | 2 | **O1:H7** | 0 | 0 | 15 | 0 | O4:NM | 0 | 0 | 1 | 0 | O88:NM | 0 | 1 | 0 | 1 |
| O105:H7 | 0 | 0 | 1 | 0 | O1:NM | 0 | 1 | 3 | 2 | O59:H21 | 0 | 0 | 0 | 1 | O89:H38 | 0 | 0 | 0 | 1 |
| O106:H18 | 1 | 0 | 0 | 0 | O2 | 0 | 0 | 7 | 0 | O5:H10 | 0 | 1 | 0 | 1 | O89:Hnt | 0 | 1 | 0 | 0 |
| O106:NM | 0 | 1 | 0 | 0 | O20:H19 | 0 | 1 | 0 | 0 | O5:Hnt | 0 | 1 | 0 | 0 | O8:H19 | 0 | 0 | 0 | 1 |
| O112:H8 | 0 | 0 | 0 | 2 | O21:H4 | 0 | 0 | 2 | 0 | O5:NM | 1 | 2 | 0 | 0 | O8:H4 | 0 | 0 | 0 | 1 |
| O113:H21 | 0 | 0 | 0 | 1 | O21:NM | 0 | 0 | 0 | 1 | O6 | 0 | 2 | 3 | 0 | O8:Hnt | 0 | 2 | 0 | 0 |
| O114:NM | 0 | 0 | 0 | 1 | O22 | 0 | 1 | 0 | 0 | **O6:H1** | 0 | 0 | 13 | 0 | O8:NM | 0 | 2 | 1 | 1 |
| O119 | 0 | 1 | 0 | 0 | O22:H1 | 0 | 0 | 3 | 0 | O6:H10 | 0 | 2 | 0 | 0 | O92:H31 | 0 | 0 | 0 | 1 |
| O12 | 0 | 3 | 1 | 0 | O22:H6 | 0 | 0 | 1 | 0 | O6:H31 | 0 | 0 | 4 | 0 | O9:H19 | 0 | 1 | 0 | 0 |
| O121:Hnt | 0 | 1 | 0 | 0 | O22:Hnt | 0 | 0 | 1 | 0 | O6:H7 | 0 | 0 | 2 | 0 | O9:NM | 0 | 1 | 0 | 1 |
| O125:H10 | 0 | 0 | 1 | 0 | O23:H15 | 0 | 0 | 0 | 1 | O6:NM | 0 | 0 | 8 | 0 | OM:H18 | 1 | 0 | 0 | 0 |
| O12:H5 | 0 | 0 | 1 | 0 | O24:H4 | 0 | 0 | 0 | 3 | O7 | 1 | 0 | 0 | 0 | Ont:H1 | 0 | 0 | 8 | 0 |
| O131:H25 | 1 | 0 | 0 | 0 | O24:H4 | 0 | 0 | 0 | 1 | O74:H1 | 0 | 0 | 3 | 0 | Ont:H10 | 0 | 1 | 1 | 0 |
| O131:NM | 0 | 1 | 0 | 0 | O25:H1 | 0 | 0 | 5 | 0 | O74:H6 | 0 | 0 | 6 | 0 | Ont:H14 | 0 | 0 | 0 | 1 |
| O136:NM | 0 | 0 | 2 | 0 | O25:H31 | 0 | 0 | 1 | 0 | O74:NM | 0 | 0 | 2 | 0 | Ont:H16 | 0 | 0 | 0 | 1 |
| O13:H4 | 0 | 0 | 1 | 0 | O25:H4 | 0 | 0 | 1 | 0 | O75:H5 | 0 | 0 | 2 | 0 | Ont:H18 | 1 | 0 | 1 | 0 |
| O144:H8 | 0 | 1 | 0 | 0 | O25:HN | 0 | 0 | 1 | 0 | O75:H7 | 0 | 0 | 3 | 0 | Ont:H26 | 1 | 0 | 0 | 0 |
| O145:H31 | 0 | 0 | 2 | 0 | O25:Hnt | 0 | 0 | 1 | 0 | O75:Hnt | 0 | 0 | 1 | 0 | Ont:H31 | 0 | 0 | 1 | 0 |
| O147 | 0 | 0 | 1 | 0 | O25:NM | 0 | 1 | 1 | 0 | O77:H18 | 2 | 0 | 0 | 0 | Ont:H32 | 0 | 1 | 0 | 0 |
| O149 | 0 | 1 | 0 | 0 | O26:H32 | 0 | 1 | 0 | 0 | O77:H34 | 0 | 1 | 0 | 0 | Ont:H4 | 0 | 2 | 2 | 0 |
| O150:H21 | 0 | 0 | 0 | 1 | O26:H7 | 0 | 1 | 0 | 0 | O78 | 0 | 4 | 0 | 1 | Ont:H5 | 0 | 1 | 2 | 1 |
| O152:H10 | 0 | 0 | 1 | 0 | O29:H51 | 0 | 0 | 0 | 1 | O78:H4 | 0 | 0 | 0 | 2 | Ont:H6 | 0 | 2 | 2 | 1 |
| O153:H34 | 2 | 0 | 0 | 0 | O2:H1 | 0 | 0 | 2 | 0 | O78:H51 | 0 | 0 | 0 | 3 | Ont:H7 | 0 | 0 | 6 | 0 |
| O153:H6 | 0 | 0 | 0 | 1 | O2:H4 | 0 | 0 | 3 | 3 | O78:H9 | 0 | 1 | 0 | 0 | Ont:Hnt | 3 | 10 | 6 | 1 |
| O159:H28 | 0 | 0 | 0 | 1 | **O2:H5** | 0 | 0 | 10 | 0 | **O78:NM** | 0 | 13 | 1 | 0 | Ont:NM | 2 | 6 | 4 | 5 |
| O15:H10 | 0 | 0 | 0 | 1 | O2:H6 | 0 | 1 | 5 | 0 | O79:H25 | 0 | 0 | 0 | 1 | Orough:H1 | 0 | 0 | 2 | 0 |
| O15:H24 | 1 | 1 | 0 | 0 | O2:H7 | 0 | 0 | 2 | 0 | O79:H43 | 1 | 0 | 0 | 0 | Orough:H18 | 0 | 0 | 0 | 1 |
| O15:NM | 0 | 1 | 0 | 0 | O2:H9 | 0 | 0 | 0 | 2 | O79:NM | 0 | 1 | 0 | 0 | Orough:H4 | 0 | 0 | 0 | 1 |
| O16 | 0 | 1 | 0 | 0 | O2:Hnt | 0 | 0 | 7 | 1 | O7:H15 | 0 | 0 | 0 | 1 | Orough:H48 | 0 | 0 | 0 | 1 |
| O166:H15 | 0 | 0 | 0 | 1 | O2:NM | 0 | 1 | 3 | 1 | O7:H21 | 0 | 0 | 0 | 1 | Orough:H6 | 0 | 0 | 1 | 0 |
| O16:H6 | 0 | 1 | 1 | 0 | O32 | 0 | 0 | 1 | 0 | O7:H32 | 0 | 1 | 0 | 0 | Orough:H7 | 0 | 0 | 1 | 0 |
| O18 | 0 | 0 | 4 | 0 | O32:H34 | 0 | 1 | 0 | 0 | O7:H7 | 0 | 0 | 1 | 0 | Orough:Hnt | 0 | 1 | 0 | 0 |
| O18:H1 | 0 | 0 | 2 | 0 | O35 | 0 | 0 | 1 | 0 | O7:NM | 9 | 0 | 0 | 0 | Orough:H11 | 0 | 0 | 1 | 0 |
| O18:H4 | 0 | 1 | 0 | 0 | O43:H2 | 0 | 0 | 0 | 1 | O83:H31 | 0 | 0 | 1 | 0 | Orough:H31 | 0 | 0 | 3 | 0 |
| O18:H5 | 0 | 0 | 8 | 0 | O45 | 1 | 0 | 2 | 0 | O85:Hnt | 0 | 0 | 0 | 1 |  |  |  |  |  |
| O18:H6 | 0 | 0 | 3 | 0 | O46:NM | 0 | 0 | 1 | 0 | O86:H18 | 0 | 0 | 0 | 2 |  |  |  |  |  |

*o454-nlpD* patterns: I = *o454*-negative, II = 1.319 bp, III = 3.685 bp, IV = 4.546 bp

Ont: O antigen not typable, although tested; Hnt: H antigen not typable, although tested; NM: non-motile. The most common serogroups are reported with bold character.
