# Supplementary material for: Correlation between the genomic o454-nlpD region polymorphisms, virulence gene equipment and phylogenetic group of extraintestinal Escherichia coli (ExPEC) enables pathotyping irrespective of host, disease and source of isolation
Source: Gut Pathog. 2014 Sep 16;6:37. doi: 10.1186/s13099-014-0037-x (PMC4209514; doi:10.1186/s13099-014-0037-x)
Supplement: Additional file 5: — Contingency tables showing the MLST frequencies given the occurrence of theo454-nlpDpattern. The most common (n >10) sequence types (STs) are reported with bold characters. Table captions: o454-nlpD patterns: I = o454-negative, II = 1.319 bp, III = 3.685 bp, IV = 4.546 bp. [file s13099-014-0037-x-S5.docx]

Additional File 5: Contingency tables showing the MLST frequencies given the occurrence of the *o454-nlpD* pattern. The most common (n >10) sequence types (STs) are reported with bold characters.

|  | ***o454-nlpD* pattern** | | | |  | ***o454-nlpD* pattern** | | | |  | ***o454-nlpD* pattern** | | | |
| --- | --- | --- | --- | --- | --- | --- | --- | --- | --- | --- | --- | --- | --- | --- |
| **ST** | **I** | **II** | **III** | **IV** | **ST** | **I** | **II** | **III** | **IV** | **ST** | **I** | **II** | **III** | **IV** |
| **10** | 1 | 41 | 0 | 0 | 69 | 5 | 0 | 0 | 0 | 130 | 1 | 0 | 0 | 0 |
| **12** | 0 | 0 | 20 | 0 | 70 | 0 | 0 | 0 | 2 | 131 | 0 | 0 | 2 | 0 |
| 14 | 0 | 0 | 2 | 0 | 71 | 0 | 0 | 0 | 1 | 135 | 0 | 0 | 2 | 0 |
| **23** | 0 | 14 | 0 | 0 | 72 | 0 | 0 | 0 | 1 | 140 | 0 | 0 | 9 | 0 |
| 34 | 0 | 1 | 0 | 0 | **73** | 0 | 0 | 43 | 0 | **141** | 0 | 0 | 16 | 0 |
| 38 | 0 | 0 | 0 | 6 | 74 | 0 | 0 | 1 | 0 | 144 | 0 | 0 | 2 | 0 |
| 43 | 0 | 1 | 0 | 0 | 75 | 0 | 0 | 0 | 2 | 155 | 0 | 0 | 0 | 3 |
| 44 | 0 | 1 | 0 | 0 | 76 | 0 | 0 | 1 | 0 | 156 | 0 | 0 | 0 | 2 |
| 45 | 0 | 1 | 0 | 0 | 78 | 0 | 0 | 1 | 0 | 162 | 0 | 0 | 0 | 2 |
| 46 | 0 | 2 | 0 | 0 | 79 | 0 | 0 | 1 | 0 | 167 | 0 | 2 | 0 | 0 |
| 47 | 0 | 1 | 0 | 0 | 80 | 0 | 0 | 4 | 0 | 216 | 0 | 1 | 0 | 0 |
| 48 | 0 | 6 | 0 | 0 | 82 | 0 | 0 | 1 | 0 | 224 | 0 | 1 | 0 | 0 |
| 49 | 0 | 1 | 0 | 0 | 83 | 0 | 0 | 1 | 0 | 295 | 0 | 0 | 0 | 1 |
| 50 | 0 | 1 | 0 | 0 | 84 | 0 | 0 | 0 | 1 | 297 | 0 | 0 | 0 | 1 |
| 52 | 0 | 1 | 0 | 0 | 85 | 0 | 0 | 0 | 1 | 347 | 0 | 1 | 0 | 0 |
| 53 | 0 | 0 | 0 | 2 | 86 | 0 | 0 | 0 | 1 | 348 | 0 | 0 | 0 | 1 |
| 54 | 0 | 0 | 0 | 1 | 87 | 0 | 0 | 0 | 1 | 349 | 0 | 0 | 0 | 2 |
| 55 | 0 | 0 | 0 | 1 | 88 | 0 | 16 | 0 | 0 | 350 | 2 | 0 | 0 | 0 |
| 56 | 0 | 0 | 0 | 2 | 89 | 0 | 1 | 0 | 0 | 351 | 0 | 0 | 0 | 1 |
| 57 | 2 | 0 | 0 | 0 | 92 | 0 | 0 | 1 | 0 | 352 | 0 | 0 | 1 | 0 |
| 58 | 0 | 0 | 0 | 2 | 93 | 0 | 4 | 0 | 0 | 353 | 0 | 0 | 1 | 0 |
| 59 | 0 | 0 | 0 | 2 | **95** | 0 | 0 | 68 | 0 | 354 | 4 | 0 | 0 | 0 |
| 60 | 0 | 0 | 0 | 1 | 100 | 0 | 1 | 0 | 0 | 355 | 0 | 0 | 2 | 0 |
| 61 | 1 | 0 | 0 | 0 | 101 | 0 | 0 | 0 | 2 | 356 | 0 | 1 | 0 | 0 |
| 62 | 10 | 0 | 0 | 0 | 104 | 0 | 0 | 3 | 0 | 357 | 0 | 0 | 1 | 0 |
| 63 | 0 | 1 | 0 | 0 | 115 | 0 | 0 | 0 | 4 | 358 | 0 | 0 | 2 | 0 |
| 64 | 1 | 0 | 0 | 0 | **117** | 0 | 0 | 0 | 15 | 359 | 0 | 0 | 0 | 2 |
| 65 | 1 | 0 | 0 | 0 | 118 | 0 | 1 | 0 | 0 | 361 | 0 | 1 | 0 | 0 |
| 66 | 1 | 0 | 0 | 0 | 125 | 0 | 0 | 0 | 1 | 362 | 1 | 0 | 0 | 0 |
| 67 | 0 | 0 | 0 | 1 | 126 | 0 | 0 | 2 | 0 | 363 | 0 | 0 | 2 | 0 |
| 68 | 0 | 0 | 0 | 4 | 127 | 0 | 1 | **12** | 0 | 365 | 0 | 0 | 0 | 1 |

*o454-nlpD* patterns: I = *o454*-negative, II = 1.319 bp, III = 3.685 bp, IV = 4.546 bp
