# Supplementary material for: Correlation between the genomic o454-nlpD region polymorphisms, virulence gene equipment and phylogenetic group of extraintestinal Escherichia coli (ExPEC) enables pathotyping irrespective of host, disease and source of isolation
Source: Gut Pathog. 2014 Sep 16;6:37. doi: 10.1186/s13099-014-0037-x (PMC4209514; doi:10.1186/s13099-014-0037-x)
Supplement: Additional file 6: — Contingency tables showing the Ecor frequencies given the occurrence of theo454-nlpDpattern. Table captions: o454-nlpD patterns: I = o454-negative, II = 1.319 bp, III = 3.685 bp, IV = 4.546 bp. [file s13099-014-0037-x-S6.docx]

Additional File 6: Contingency tables showing the Ecor frequencies given the occurrence of the *o454-nlpD* pattern

| **Ecor group** | ***o454-nlpD* pattern** | | | |
| --- | --- | --- | --- | --- |
|  | **I** | **II** | **III** | **IV** |
| A | 1 | 69 | 0 | 1 |
| ABD | 24 | 3 | 2 | 24 |
| AxB1 | 2 | 10 | 0 | 27 |
| B1 | 0 | 39 | 0 | 21 |
| B2 | 0 | 1 | 249 | 0 |
| D | 14 | 2 | 0 | 21 |

*o454-nlpD* patterns: I = *o454*-negative, II = 1.319 bp, III = 3.685 bp, IV = 4.546 bp
