# Supplementary material for: Correlation between the genomic o454-nlpD region polymorphisms, virulence gene equipment and phylogenetic group of extraintestinal Escherichia coli (ExPEC) enables pathotyping irrespective of host, disease and source of isolation
Source: Gut Pathog. 2014 Sep 16;6:37. doi: 10.1186/s13099-014-0037-x (PMC4209514; doi:10.1186/s13099-014-0037-x)
Supplement: Additional file 7: — Contingency tables showing the Class/Pathotype frequencies given the occurrence of theo454-nlpDpattern. Table captions: o454-nlpD patterns: I = o454-negative, II = 1.319 bp, III = 3.685 bp, IV = 4.546 bp. [file s13099-014-0037-x-S7.docx]

Additional File 7: Contingency tables showing the Class/Pathotype frequencies given the occurrence of the *o454-nlpD* pattern

| **Class/Pathotype** | ***o454-nlpD* pattern** | | | |
| --- | --- | --- | --- | --- |
|  | **I** | **II** | **III** | **IV** |
| APEC | 4 | 34 | 65 | 32 |
| Commensal | 14 | 43 | 53 | 33 |
| ExPEC | 3 | 4 | 2 | 9 |
| NMEC | 5 | 1 | 17 | 1 |
| SePEC | 4 | 13 | 10 | 5 |
| UPEC | 11 | 29 | 104 | 14 |

*o454-nlpD* patterns: I = *o454*-negative, II = 1.319 bp, III = 3.685 bp, IV = 4.546 bp
