# Supplementary material for: Correlation between the genomic o454-nlpD region polymorphisms, virulence gene equipment and phylogenetic group of extraintestinal Escherichia coli (ExPEC) enables pathotyping irrespective of host, disease and source of isolation
Source: Gut Pathog. 2014 Sep 16;6:37. doi: 10.1186/s13099-014-0037-x (PMC4209514; doi:10.1186/s13099-014-0037-x)
Supplement: Additional file 8: — Contingency tables showing the host species frequencies given the occurrence of theo454-nlpDpattern. Table captions: o454-nlpD patterns: I = o454-negative, II = 1.319 bp, III = 3.685 bp, IV = 4.546 bp. [file s13099-014-0037-x-S8.docx]

Additional File 8: Contingency tables showing the host species frequencies given the occurrence of the *o454-nlpD* pattern

| **Host species** | ***o454-nlpD* pattern** | | | |
| --- | --- | --- | --- | --- |
|  | **I** | **II** | **III** | **IV** |
| Bird | 6 | 36 | 84 | 40 |
| Bison | 0 | 0 | 0 | 1 |
| Bovine | 0 | 3 | 0 | 0 |
| Cat | 2 | 2 | 27 | 2 |
| Cougar | 1 | 0 | 0 | 0 |
| Dog | 2 | 11 | 39 | 5 |
| Giraffe | 0 | 0 | 0 | 2 |
| Goat | 0 | 0 | 0 | 1 |
| Horse | 3 | 7 | 2 | 8 |
| Human | 23 | 59 | 95 | 22 |
| Kangaroo rat | 0 | 0 | 0 | 1 |
| Leopard | 1 | 1 | 0 | 0 |
| Lion | 0 | 0 | 0 | 1 |
| Pig | 1 | 2 | 0 | 7 |
| Primates | 1 | 3 | 4 | 3 |
| Sheep | 1 | 0 | 0 | 1 |

*o454-nlpD* patterns: I = *o454*-negative, II = 1.319 bp, III = 3.685 bp, IV = 4.546 bp
